# Whole-genome methylation profiling of the retinal pigment epithelium of individuals with age-related macular degeneration reveals differential methylation of the *SKI*, *GTF2H4*, and *TNXB* genes

**DOI:** 10.1186/s13148-019-0608-2

**Published:** 2019-01-14

**Authors:** Louise F. Porter, Neil Saptarshi, Yongxiang Fang, Sonika Rathi, Anneke I. den Hollander, Eiko K. de Jong, Simon J. Clark, Paul N. Bishop, Timothy W. Olsen, Triantafillos Liloglou, Venkata R. M. Chavali, Luminita Paraoan

**Affiliations:** 1 St Paul’s Eye Unit, Royal Liverpool University Hospital, Liverpool, UK; 2 Department of Eye and Vision Science, William Duncan Building, University of Liverpool, Liverpool, UK; 3 Centre for Genomic Research, University of Liverpool, Liverpool, UK; 4 Institute of Translational Medicine, University of Liverpool, Liverpool, UK; 5 Department of Ophthalmology, University of Pennsylvania School of Medicine, Philadelphia, USA; 6 Department of Ophthalmology, Donders Institute for Brain, Cognition and Behaviour, Radboud University Medical Center, Nijmegen, The Netherlands; 7 Division of Evolution and Genomic Sciences, School of Biological Sciences, Faculty of Biology, Medicine and Health, University of Manchester, Manchester, UK; 8 Manchester Royal Eye Hospital, Manchester University NHS Foundation Trust, Manchester Academic Health Science Centre, Manchester, UK; 9 Mayo Clinic, Rochester, MN USA

## Abstract

**Background:**

Age-related macular degeneration (AMD) is a degenerative disorder of the central retina and the foremost cause of blindness. The retinal pigment epithelium (RPE) is a primary site of disease pathogenesis. The genetic basis of AMD is relatively well understood; however, this knowledge is yet to yield a treatment for the most prevalent non-neovascular disease forms. Therefore, tissue-specific epigenetic mechanisms of gene regulation are of considerable interest in AMD. We aimed to identify differentially methylated genes associated with AMD in the RPE and differentiate local DNA methylation aberrations from global DNA methylation changes, as local DNA methylation changes may be more amenable to therapeutic manipulation.

**Methods:**

Epigenome-wide association study and targeted gene expression profiling were carried out in RPE cells from eyes of human donors. We performed genome-wide DNA methylation profiling (Illumina 450k BeadChip array) on RPE cells from 44 human donor eyes (25 AMD and 19 normal controls). We validated the findings using bisulfite pyrosequencing in 55 RPE samples (30 AMD and 25 normal controls) including technical (*n* = 38) and independent replicate samples (*n* = 17). Long interspersed nucleotide element 1 (LINE-1) analysis was then applied to assess global DNA methylation changes in the RPE. RT-qPCR on independent donor RPE samples was performed to assess gene expression changes.

**Results:**

Genome-wide DNA methylation profiling identified differential methylation of multiple loci including the SKI proto-oncogene (*SKI*) (*p* = 1.18 × 10^−9^), general transcription factor IIH subunit H4 (*GTF2H4) (p =* 7.03 × 10^−7^), and Tenascin X (*TNXB*) (*p* = 6.30 × 10^−6^) genes in AMD. Bisulfite pyrosequencing validated the differentially methylated locus cg18934822 in *SKI*, and cg22508626 within *GTF2H4*, and excluded global DNA methylation changes in the RPE in AMD. We further demonstrated the differential expression of *SKI*, *GTF2H4*, and *TNXB* in the RPE of independent AMD donors.

**Conclusions:**

We report the largest genome-wide methylation analysis of RPE in AMD along with associated gene expression changes to date, for the first-time reaching genome-wide significance, and identified novel targets for functional and future therapeutic intervention studies. The novel differentially methylated genes *SKI* and *GTF2H4* have not been previously associated with AMD, and regulate disease pathways implicated in AMD, including TGF beta signaling (*SKI*) and transcription-dependent DNA repair mechanisms (*GTF2H4*).

**Electronic supplementary material:**

The online version of this article (10.1186/s13148-019-0608-2) contains supplementary material, which is available to authorized users.

## Background

Age-related macular degeneration (AMD) is a degenerative disorder of the central retina and the most common cause of sight impairment in those aged over 50 [1]. It is predicted to affect 288 million people by 2040 [2]. AMD progresses from early and intermediate forms through to late AMD, which manifests as choroidal neovascularization (CNV) and/or geographic atrophy (GA). Whilst CNV (“wet” AMD) is treatable, currently, there is no treatment for non-neovascular AMD forms. Early and intermediate AMD are characterized by the accumulation of medium size (between 63 and 125 μm) extracellular lipo-proteinaceous deposits termed drusen, located between the inner collagenous layers of Bruch’s membrane and the basement membrane of the retinal pigment epithelium (RPE), pigmentary abnormalities, and progressive photoreceptor dysfunction at the macula [3]. The genetics of AMD has been investigated extensively and clinical trials based on genome-wide association study (GWAS) candidate genes have emerged [4–6]; however, despite the success of genetic studies, causal and functional effects of AMD risk variants are not understood. Recently a clinical trial targeting the complement pathway failed to meet the primary study endpoint (https://clinicaltrials.gov/ct2/show/NCT02247531). Environmental risk factors associated with oxidative stress have been identified, including smoking, dietary fat, omega-3 fatty acid, and antioxidant intake, although it is unclear how these factors contribute to the mechanisms of disease in AMD [1].

Epigenetic dysfunction of the RPE, a primary site of AMD pathogenesis, has been suggested to drive early disease following the finding of a widespread decrease of chromatin accessibility in AMD [7]. Tissue-specific DNA methylation may underlie the poorly understood interplay of genetic and environmental risk factors [8] and several groups reported that DNA methylation changes in individual genes may be associated with AMD [9–13]. However, in these studies, candidate loci did not reach genome-wide significance, and they were hampered by small sample sizes of relevant tissues and the use of peripheral blood as a proxy.

In this study, we investigated DNA methylation changes in AMD by performing genome-wide DNA methylation profiling in RPE cells of human donors (44 samples; 25 AMD and 19 normal) to investigate whether DNA methylation underlies differential gene expression in AMD.

## Methods

### Sample collection, grading, and DNA extraction

Ocular tissue for DNA methylation analyses (Illumina 450k BeadChip array and pyrosequencing) was obtained from the Manchester Eye Bank, UK. DNA was extracted between 24 and 36 h post-mortem from individuals aged over 50 years. Demographic information obtained included sex, age, cause and time of death, time of enucleation, ocular history where possible (from General Practitioner NHS summary sheets enclosed with the eye donation consent forms), and any major medical conditions. Eye dissection was performed under a GT Vision GXM XTL3TV6 stereomicroscope fitted with a GXCAM 5 digital color camera used to take high-resolution macular photographs. Photographs for grading were reviewed by one (LFP) and where uncertainty was present two ophthalmologists (LFP and PB). RPE cells were isolated from Bruch’s mechanically, by scraping with a 22-gauge needle and removal with a silicone brush. DNA was isolated from harvested RPE cells using a DNA purification kit and bisulfite conversion was performed using the Zymo EZ DNA methylation tissue kit (Zymo Research, Irvine, USA). Tissue phenotyping was linked to the Age-Related Eye Disease Study (AREDS) classification, using key features as described in the Minnesota Grading System [14, 15] (MGS). We subdivided the tissue into no AMD (no drusen or a few small drusen < 63 μm) and AMD, based upon the criteria for intermediate drusen (> 63 μm and < 125 μm), or the presence of one or more large drusen (> 125 μm), and the presence of pigmentary changes or geographic atrophy (Additional file 1: Table S1 and S2). We deliberately excluded samples with CNV (MGS level 4) for the purposes of this study. The purity of RPE cells for methylation studies was confirmed by measuring RPE, retinal, neuronal, and choroidal markers in our in-house RNA sequencing data. Analysis of DNA degradation levels prior to methylation analyses was performed by gel-electrophoresis in the manner described by Rhein et al., (2015) [16].

The RPE samples used for expression studies (qRT-PCR and RNA sequencing) were obtained by Lions Eye Institute of Transplant and Research (LEITR), Florida, USA [17]. After necessary pictures were obtained for grading, the RPE tissues were brushed off, collected, and stored in RNA later solution within a post-mortem time of 6 h. Normal or AMD status was assigned by MGS [14, 15] by an ophthalmologist (TWO) (Additional file 1: Table S1 and S2).

### Illumina Infinium Human Methylation 450k BeadChip Array

DNA methylation levels were measured using the Illumina Infinium HumanMethylation450 BeadChip Array platform (Illumina, Inc., San Diego, CA, USA) that interrogates over 450,000 CpG sites representing about 99% of the RefSeq genes. Four microliters of bisulfite converted DNA was processed and hybridized to the microarray according to the manufacturer’s protocol. Analysis was performed at GenomeScan (GenomeScan B.V., Leiden, the Netherlands). All samples were run within a single array with technical controls incorporated into the experimental design and a duplicate sample run. Data has been deposited in ArrayExpress with submission number E-MTAB-7183.

Quality control was conducted in GenomeStudio software according to the manufacturer’s instructions, assessed using the R script MethylAid with no initial normalization. Data analysis was performed in R (version 3.3.1) integrating “minfi” [18], “DMRcate” [19], “limma” [20], “missMethyl” [21], and “IlluminaHumanMethylation450kanno.ilmn12.hg1” using the *M* metric raw methylation values. Data were further inspected for quality metrics, adjusted for color channel imbalance, and quantile-normalized using SWANN normalization. In the DML analysis, the sample’s sex origin was predicted using the R function “getSex.” Data sets were filtered to remove probes from chromosomes X and Y, probes known to be cross-reactive https://www.ncbi.nlm.nih.gov/pmc/articles/PMC3592906/ (supplementary material), and probes on which poor quality data were generated (detection *p* values ≥ 0.01). We removed probes associated with SNPs of minor allele frequency (MAF) ≥ 0.05 using the function “dropLociWithSnps.” A linear model-based DML analysis was applied using the *M* values of filtered data and seven parameters listed in Additional file 1: Table S3. Log2 FC (odds ratio of the fold change) of *M* values for each contrast were computed and tested using *t* tests for associated *p* values. *p* values were adjusted for multiple testing using the FDR approach [22]. Significantly differential expression was defined as those with an FDR-adjusted *p* value < 10%. If no DML were detected or the number of DML was less than 10, a *p* value cutoff of *p* ≤ 0.0001 was used. Multidimensional scaling (MDS) plots, correlation heatmaps, and PCA (principal component analysis) plots were employed to visualize the data variation.

Differentially methylated region (DMR) analysis was performed using DMRcate from the R package DMPcate. A list of top CpGs was generated based on a relaxed threshold *p* value ≤ 0.0001. The process was carried out on AMD vs normal and interaction model parameters only.

We also applied the “GapHunter” algorithm in R to our 450k array dataset to “flag” probes that displayed *β* value distributions containing discrete clusters [23, 24].

### Bisulfite pyrosequencing of candidate differentially methylated positions

Bisulfite-pyrosequencing is an established technique in single-nucleotide resolution, quantitative methylation analysis of genomic regions [25]. Assays were designed using PyroMark Assay Design Software (Version 2.0.1) to interrogate regions containing or surrounding (< 100 bp upstream/downstream) candidate DML identified in our Illumina Human Methylation 450k BeadChip. The percent methylation for each CpG site within the target sequence was calculated using the PyroQCpG Software (Qiagen) with non-CpG cytosine residues used as built-in controls to verify complete bisulfite conversion. Genomic DNA was bisulfite converted using the EZ DNA Methylation-Gold Kit (Zymo Research, Irvine, USA). Due to limited amounts of starting DNA, we employed “Quantitative Methylation Analysis of Minute DNA Amounts After Whole Bisulfitome Amplification”, known as qMAMBA [26, 27]. DNA Whole Genome Amplification (WGA) was carried out on bisulfite-modified RPE gDNA using the Repli-G Screening Kit (Qiagen, Hilden, Germany) as per the manufacturer’s instructions. PCR amplicons were designed using PyroMark Assay Design SW 2.0 software (Qiagen, Hilden, Germany) (Additional file 1: Table S4) with a methylation standard curve used ensure the assay was without methylation bias [25]. Robust PCR end-products were immobilized to suspended Streptavidin Sepharose™ High-Performance beads (GE Healthcare Life Sciences, Pittsburgh, Pennsylvania, USA) and single-strand DNA (ssDNA) template captured from candidate gene amplicons [25]. Sequencing primers (10 pmol; Eurofins Genomics, Edelberg, Germany) were subsequently annealed to the ssDNA template and pyrosequenced using a PyroMark-Q96 ID as per manufacturer’s instructions. All pyrosequencing data were analyzed using CpG run-analysis on PyroMark Q96 (version 2. 5. 8). Mean methylation was calculated for ≥ 4 CpGs per genomic region with Mann-Whitney *U* test used to compare differences in mean-methylation between AMD and normal patients. All statistical analysis was carried out using GraphPad Prism (Version 7.0, GraphPad Software Inc., San Diego, CA, USA). Six samples belonging to the DNA methylation array cohort (epigenome-wide association study (EWAS) cohort) were excluded from bisulfite-pyrosequencing due to limited DNA availability, DNA degradation, or poor-quality PCR-amplification. The final cohort comprised 55 RPE patient and normal samples, with no significant age differences detected (Additional file 1: Table S5). Pooled and sex-stratified analyses were performed.

### Quantitative real-time PCR (qRT-PCR) of differentially methylated genes

Expression levels of candidate differentially methylated genes identified from our array, *GTF2H4*, *SKI*, *RIC3*, *EIF2AK3*, *GRIA4*, and DMR gene *TNXB* were determined by qRT-PCR (delta delta Ct) using 7900HT Fast Real-Time PCR (Thermofisher Scientific, MA, USA). Total RNA was extracted from RPE isolated from four AMD-affected donors (excluding CNV) and three normal donors (Additional file 1: Table S2) from LEITR, Florida, using Direct–Zol RNA MiniPrep kit (Zymo Research, CA, USA). Two micrograms of total RNA was reverse transcribed into cDNA using Superscript III First-Strand cDNA Synthesis (cat no. 18080-051, Thermo Fisher Scientific, MA, USA) and equal amounts of cDNA (30 ng) amplified using Power Up SYBR Green with gene-specific primers (Additional file 1: Table S6). *GAPDH* was included as an endogenous control. Fold change was calculated between different subgroups using the delta delta Ct method [28] and significantly differentially expressed genes were identified based on a *p* value< 0.05. Experiments were performed in triplicate and data pooled to generate normal versus AMD expression changes.

## Results

### Differentially methylated loci identified in AMD using the Illumina Infinium Human Methylation 450k BeadChip array

We performed an EWAS using the Illumina Human Methylation 450k BeadChip array (450k array) to investigate a role for DNA methylation in AMD using RPE samples from AMD donors (*n* = 25) and normal control donors (*n* = 19) from the Manchester Eye Bank (Additional file 1: Table S2 and S5). All AMD samples analyzed were early (level 2) and intermediate (level 3) AMD, with level 2 samples representing 84% of the cohort analyzed and level 3 samples the remaining 16%. AMD and normal control donors had comparable demographic characteristics (Additional file 1: Table S5).

Data quality assessment based on probe signals compared to background noise and quality control report function [18] concluded acceptable data quality for inclusion in downstream analyses (Additional file 2: Figure S1, S2, S3). MDS analysis indicated that sex was the strongest discriminating factor.

We employed linear interaction modeling considering sex-related methylation differences, batch effect, and disease state, comparing AMD to normal RPE samples. We identified 11 differentially methylated loci (DML) with an FDR < 20% (Table 1). We then applied a mean methylation difference cutoff (Δ*β*) of 10% to the methylation dataset (FDR ≤ 0.2, Δ*β* ≥ 0.1 (10% methylation difference)) identifying 8 DML (Table 2). Of the 8 DML, 3 candidate CpG probes, cg18486102 (*FAIM2*, *p* = 1.08 × 10^−12^), cg18934822 (*SKI*, *p* = 1.18 × 10^−9^), and cg23169512 (*p* = 2.08 × 10^−8^), reached genome-wide significance (*p* ≤ 5 × 10^−8^).

**Table 1:** Significantly differentially methylated CpG probes identified in AMD RPE cells

Probe ID	Log2 FC	Adjusted *p* value	False discovery rate (< 0.2)	Δ*β* (* > 10%)	Chromosome	Position	Relation to CpG island	UCSC gene name	Relation to gene
cg18486102	1.81	1.08E−12	4.6E−07	*0.20	chr12	50297777	Island	*FAIM2*	TSS200
cg18934822	− 3.00	1.18E−09	2.5E−04	*− 0.11	chr1	2191402	Open Sea	*SKI*	Body
cg23169512	− 3.51	2.08E−08	2.9E−03	*− 0.33	chr15	60290666	N_Shore		
cg22508626	1.02	7.03E−07	6.0E−02	*0.12	chr6	30879905	N_Shore	*GTF2H4*	Body
cg01560972	− 3.87	2.03E−06	1.4E−01	*− 0.33	chr11	8190837	S_Shore	*RIC3*	TSS1500
cg26962595	1.18	2.75E−06	1.6E−01	0.02	chr11	72504889	Island	*STARD10*	TSS200
cg11897517	1.67	3.87E−06	1.6E−01	0.02	chr6	109761938	Island	*SMPD2*	5′UTR
cg04838987	− 1.76	3.98E−06	1.6E−01	*− 0.18	chr20	33734406	N_Shore	*EDEM2*	Body
cg11241206	− 2.82	4.44E−06	1.6E−01	*− 0.35	chr11	27723128	S_Shore	*BDNF*	TSS1500; 5′UTR
cg03611060	2.51	5.00E−06	1.6E−01	*− 0.14	chr1	59281067	Island		
cg26347887	− 2.87	5.16E−06	1.6E−01	*− 0.28	chr2	88927196	Island	*EIF2AK3*	TSS200

*Δβ* mean *β* value AMD—mean *β* value control, *TSS* transcription start site, *5′UTR* 5*′*-untranslated region, *3′-UTR* 3*′*-untranslated region, *Body* gene body, *Intragenic* intragenic region

Analysis of Illumina 450k BeadChip array data using linear interaction modeling considering independent variables (sex M/F and batch effect) and their interaction with DNA methylation. Significantly differentially methylated CpG probes in contrasts AMD/normal with a FDR of < 20% are shown. cg23169512 is approximately 10 kb from *FOXB1*, and cg03611061 is within the long intergenic non-protein coding RNA 1135 and 30 kb upstream of *JUN* proto-oncogene

**Table 2:** Significantly differentially methylated CpG probes in AMD RPE cells with FDR < 20% also showing a mean methylation difference between AMD and normal RPE cells of 10% minimum (FDR < 0.2, Δ*β* ≥ 0.1 (10%))

Probe ID	Gene	Adjusted *p* value	*Δβ*	Chromosome	CpG position	Accession reference	Probe location
cg18486102	*FAIM2*	1.08E−12	0.20	12	50297777	NM_012306	TSS200
cg18934822	*SKI*	1.18E−09	0.11	1	2191402	NM_003036	Body
cg23169512		2.08E−08	0.33	15	60290666		Intragenic
cg22508626	*GTF2H4*	7.03E−07	0.11	6	30879905	NM_001517	Body
cg01560972	*RIC3*	2.03E−06	0.33	11	8190837	NM_024557	TSS1500
cg04838987	*EDEM2*	3.98E−06	0.18	20	33734406	NM_001145025	Body
cg11241206	*BDNF*	4.44E−06	0.30	11	27723128	NM_001143813	TSS1500
cg26347887	*EIF2AK3*	5.16E−06	0.28	2	88927196	NM_004836	TSS200

*TSS* transcription start site, *5′UTR* 5*′*-untranslated region, *3′-UTR* 3*′*-untranslated region, *Body* gene body, *Intragenic* intragenic region

Candidate DML were comprised of 4 promoter, 3 gene-body, and an intragenic-based CpG probe (cg23169512, Chr15:60290666, hg19) that mapped 6.4 kb upstream of *FOXB1* transcriptional start site (TSS).

### Identification of AMD differentially methylated CpG-rich genomic regions, involving adjacent differentially methylated loci close together

**Fig. 1:**
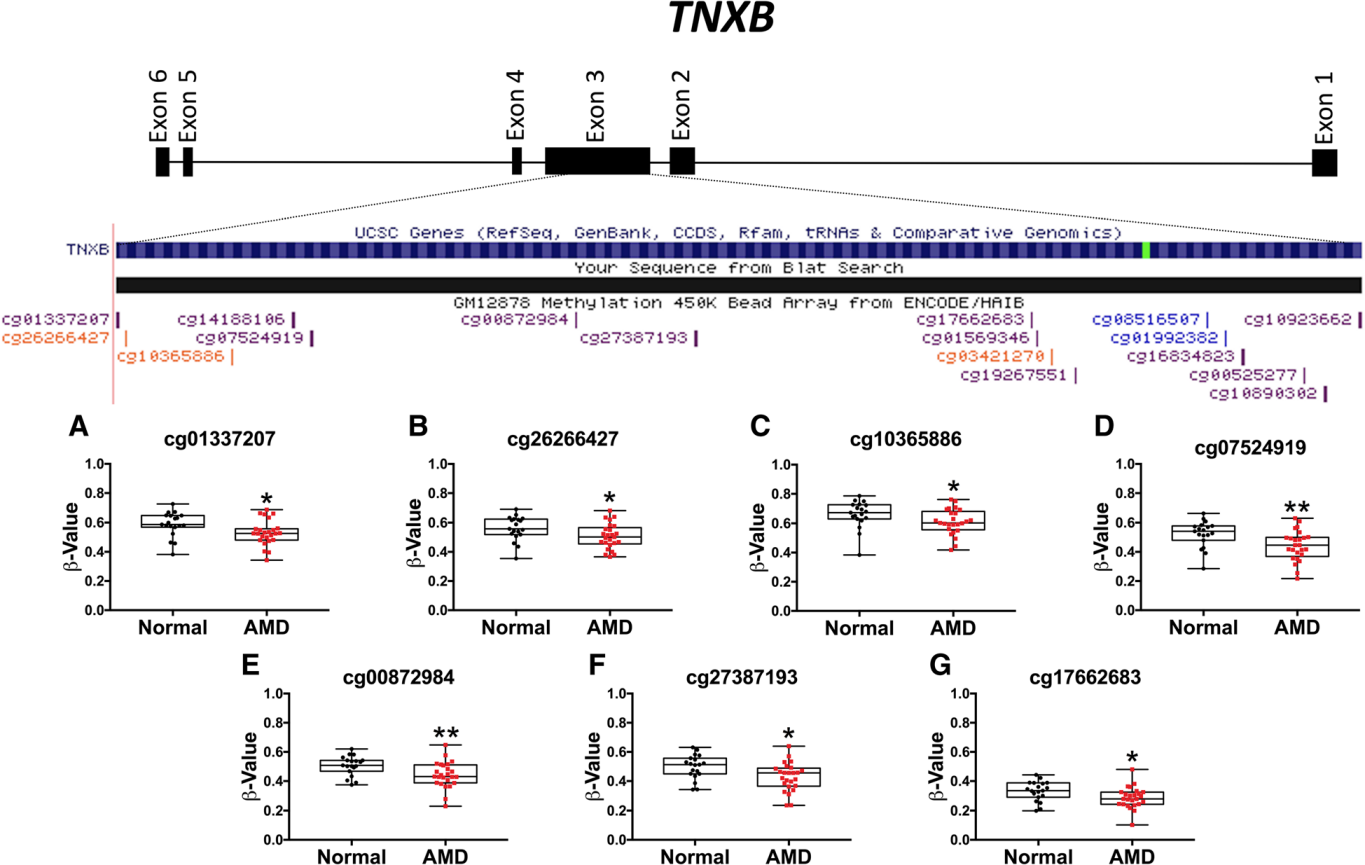
Differentially methylated region identified within tenascin X (*TNXB*). Upper panel: schematic representation of differentially methylated region (DMR) identified in the Illumina 450k BeadChip array spanning 424 bp containing 15 CpG probes within exon 3 of *TNXB*. Lower panel: univariate analyses of *β* values of seven CpG probes within the differentially methylated region. **a**–**g** Significant decrease in methylation is shown at seven CpG probes within the DMR in *TNXB* (cg01337207, *p* = 0.0204; cg26266427, *p* = 0.0361; cg10365886, *p* = 0.0248; cg07524919, *p* = 0.0065; cg00872984, *p* = 0.0088; cg27387193, *p* = 0.0191; cg17662683, *p* = 0.0233) (*p ≤ 0.05, **p ≤ 0.01). Mann-Whitney *U* test was used for statistical analyses between all groups tested

We performed DMR analysis as methylation events regulating mRNA transcription in human disease are reported to occur in CpG-rich genomic regions [29]. .As no DMRs were detected using an FDR ≤ 0.1, a *p* value cut off criterion (*P* ≤ 0.0001) was applied resulting in the identification of four DMRs (Table 3). Furthermore, two genome-wide significant DML, cg18486102 and cg18934822, mapped to two DMR within *FAIM2* and *SKI* respectively (Table 3). The fourth DMR mapping to *TNXB* (chr6:32063835-32064258, hg19) encodes the protein Tenascin X (TNX) (Fig. 1).

**Table 3:** Differentially methylated regions identified in AMD RPE cells using a relaxed *p* value cutoff criterion (*p* ≤ 0.0001)

Gene	CpG number	Chromosome coordinates	Minimum FDR	DMR Length (bp)	DMR location
*FAIM2*	7	chr12:50297477-50297945	8.39E−16	469	Promoter/exon 1/intron 1
*SKI*	6	chr1:2190850-2191658	2.92E−08	809	Intron 1
	3	chr17:14201680-14201938	3.60E−07	259	Intragenic
*TNXB*	15	chr6:32063835-32064258	6.30E−06	424	Exon 3

To examine whether the DML and DMR identified may overlap with AMD-associated genetic variants, we systematically probed the DML and DMR for the presence of one or more AMD-associated single nucleotide polymorphisms (SNP) [4]. Two variants near *TNXB* have been associated with AMD (rs12153855 and rs9391734) [30, 31] (Additional file 1: Table S7). SNP rs12153855 is located in intron 1 of *TNXB*, 10969 bp downstream of the DMR start point, and rs9391734 is 33,898 bp downstream of the DMR start point, respectively. rs429608 (associated with C2 and complement factor B [32] is 133,373 bp upstream from the DMR within *TNXB*. rs3130783, associated with *GTF2H4,* is 105,549 bp upstream of the CpG site and has also been identified as an AMD-associated disease locus [32]. These findings potentially demonstrate some overlap between the observed differences in the region-specific DNA methylation in *TNXB* and the *TNXB* AMD-associated genetic variants (Additional file 1: Table S7).

### Evaluating sex differences does not detect any sex-specific differentially methylated loci in AMD

Several studies revealed that women have a higher risk of developing AMD than men [33–35]. We analyzed the locus by locus interaction of DNA methylation levels between males (*n* = 27) and females (*n* = 17) in AMD and normal control RPE cells, to identify sex-specific differentially methylated loci using a value of FDR ≤ 0.1. We did not identify variants with a genome-wide significant sex-dependent association with AMD (*P*_sexdiff_ ≤ 5 × 10^−8^) in our systematic genome-wide search (data not shown), thereby demonstrating that there are no sexually dimorphic differentially methylated DNA loci in our dataset.

### Bisulfite pyrosequencing validates DML cg18934822 in an intronic region of *SKI* and cg22508626 in the gene body of *GTF2H4*

**Fig. 2:**
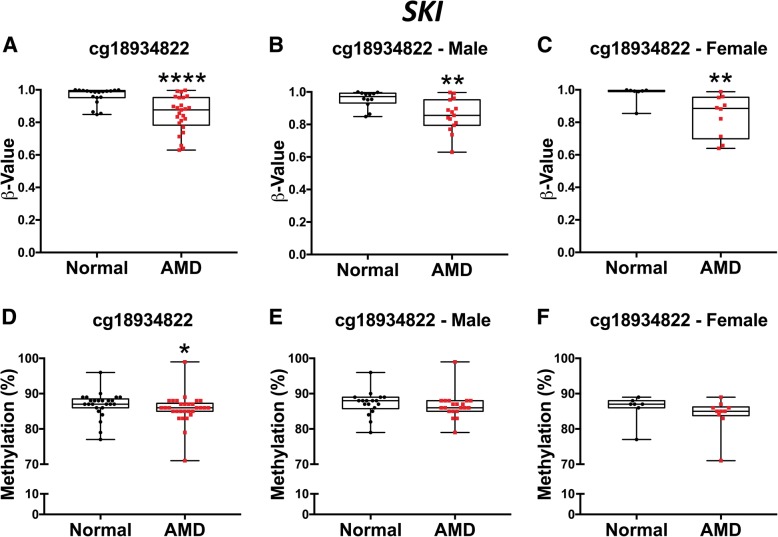
Bisulfite pyrosequencing of candidate gene *SKI* (cg18934822) validates direction of methylation change identified in 450k array. **a**–**c** Univariate analysis of *SKI* (cg18934822) in Illumina 450k BeadChip array. **a** Methylation *β* values for AMD (*n* = 25) compared to normal (*n* = 19) donor RPE cells after normalization, in addition to analysis of sex-stratified results for cg18934822 (*SKI*). Significantly reduced methylation levels are observed in AMD (*p* < 0.0001), as well as in both AMD male (*n* = 15) (**b**) and AMD female (*n* = 10) (**c**) compared to normal male (*n* = 12) (*p* = 0.0063) and normal female (*n* = 7) (*p* = 0.0031) human RPE donor cells respectively. **d**–**f** Bisulfite pyrosequencing of candidate gene *SKI* (cg18934822) in combined technical and independent sample replications. **d** Reduced methylation of cg18934822 (*SKI*) is identified in AMD (*n* = 30) compared to normal donor RPE cells (*n* = 25) (*p* = 0.0309). **e** Reduced methylation of cg18934822 (*SKI*) is identified in AMD AMD male (*n* = 20) compared to normal male (*n* = 18) (*p* = 0.1223) and **f** AMD female (*n* = 10) compared to normal female donor samples (*n* = 7) (*p* = 0.1002). (*p ≤ 0.05; **p ≤ 0.01; ***p ≤ 0.001; ****p ≤ 0.0001). All statistical analysis was performed using the Mann-Whitney *U* test

**Fig. 3:**
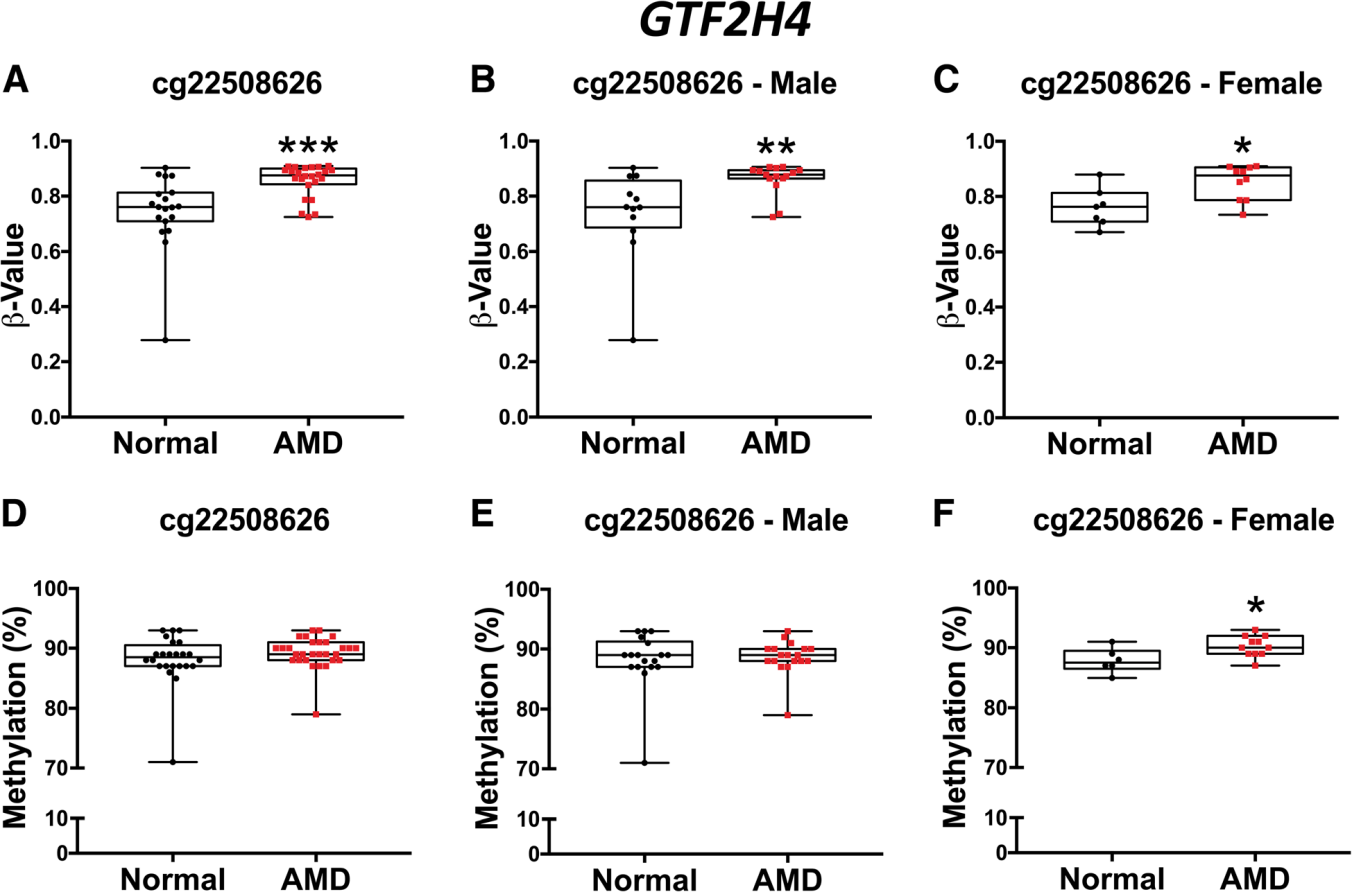
Bisulfite pyrosequencing of candidate gene *GTF2H4* (cg22508626) validates direction of methylation change identified in 450k array. **a–c** Univariate analysis of *GTF2H4* cg22508626 in Illumina 450k BeadChip array. **a** Methylation *β* values for AMD (*n* = 25) compared to normal RPE donor cells (*n* = 19) after normalization, in addition to analysis of sex-stratified results for cg22508626 (*GTF2H4*). Significantly increased methylation levels are observed in AMD compared to normal (*p* = 0.0003), in addition to both **b** AMD male (*n* = 15) and **c** AMD female (*n* = 10) compared to normal male (*n* = 12) (*p* = 0.0074) and normal female (*n* = 7) (*p* = 0.0136) human donor samples respectively. **d**–**f** Bisulfite pyrosequencing of candidate gene *GTF2H4* (cg22508626) in combined technical and independent sample replications. **d** Mean methylation difference observed in cg22508626 (*GTF2H4*) in AMD (*n* = 29) compared to normal donor samples (*n* = 24) does not reach statistical significance (*p* = 0.1263). Sex-stratified analysis reveals no significant methylation differences in **e** AMD male (*n* = 18) versus normal male (*n* = 18) donor samples. Significantly increased methylation is observed in **f** AMD female (*n* = 11) versus normal male (*n* = 6) (*p* = 0.0292). (*p ≤ 0.05)(**p ≤ 0.01)(***p ≤ 0.001)(****p ≤ 0.0001). All statistical analysis was performed using the Mann-Whitney *U* test

We then interrogated cg18934822 (*SKI*), cg22508626 (*GTF2H4*), cg26347887 (*EIF2AK3*), cg01560972 (*RIC3*), cg18486102 (*FAIM2*), and cg03243226 (*GRIA4*) in technical (*n* = 38) and biological (*n* = 17) validation sets from human RPE samples to quantify mean methylation differences between AMD and normal RPE cells (Additional file 1: Table S2 and S5). Targets were selected based on feasibility of pyrosequencing assay design, ontological relevance, transcriptomic evidence [17, 32, 36] (Additional file 1: Table S7), and FDR < 0.2, Δ*β* ± 0.1 and *p* ≤ 10^−6^. Combined cohort (*n* = 55) included 26 AMD level 2 samples (81.25%) and 6 AMD level 3 samples (18.75%). We confirmed reduced methylation for cg18934822 (*SKI*) in AMD compared to normal control donor samples (Mann-Whitney *U* test, *p* = 0.0309), in agreement with the 450k array data (Fig. 2). We also confirmed the increased methylation observed in our 450k array data for cg22508626 (*GTF2H4*, Fig. 3). This probe reached significance in sex-stratified analysis with significant hypermethylation observed in AMD in females (Mann-Whitney *U* test, *p* = 0.0292) (Fig. 3f)*.*

**Fig. 4:**
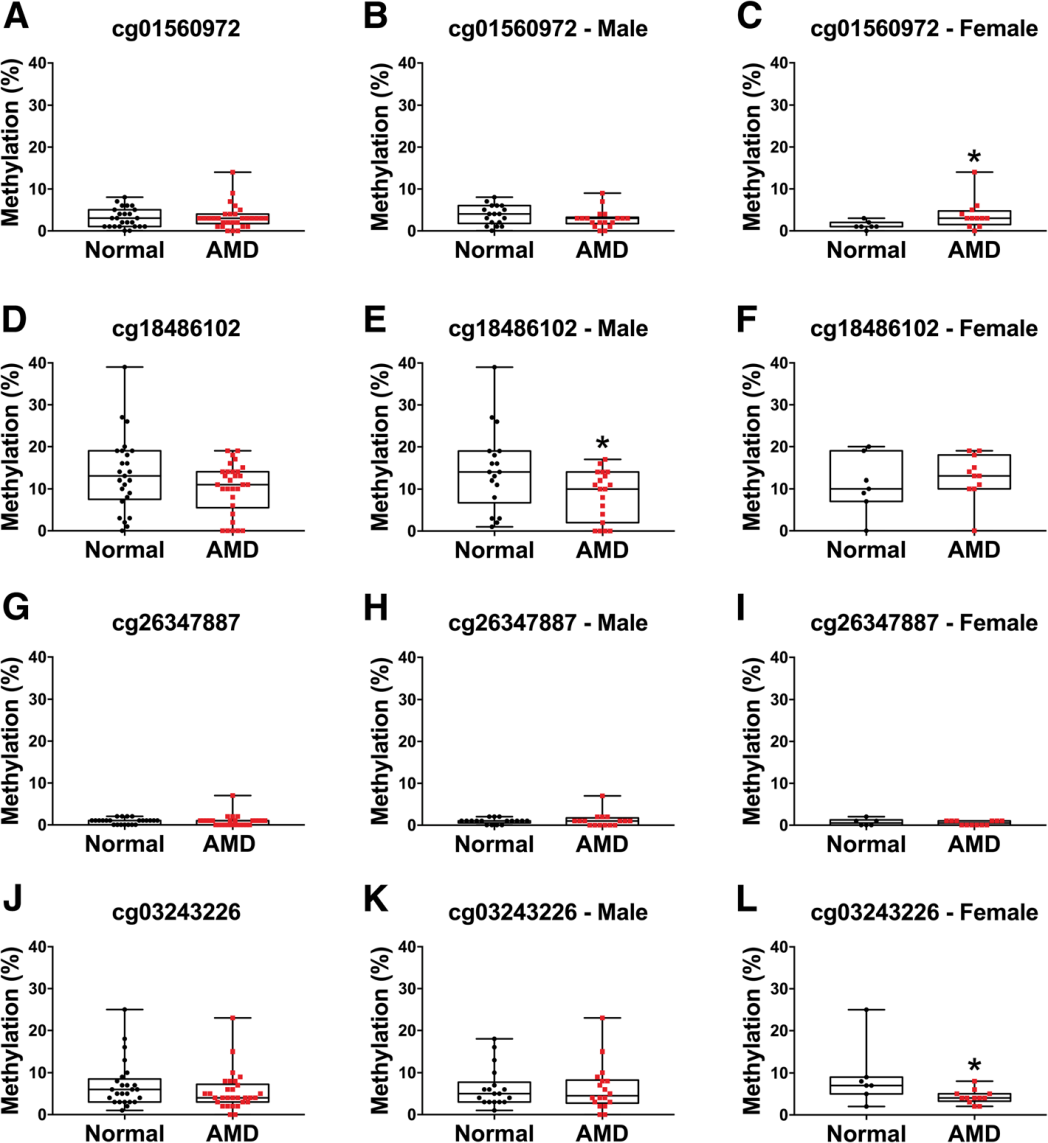
Bisulfite pyrosequencing of candidate genes: *RIC3* (cg01560972), *FAIM2* (cg18486102), *EIF2AK3* (cg26347887), and *GRIA4* (cg03243226). **a** No significant mean methylation difference was observed for cg01560972 (*RIC3*) in AMD (*n* = 30) compared to normal (*n* = 25) donor RPE cells *(p* = 0.9491). **b** Sex-stratified analysis did not reveal significant differences in AMD male (*n* = 18) compared to normal male (*n* = 18) (*p* = 0.2006). **c** A significant hypermethylation was observed in AMD female (*n* = 12) compared to normal female (*n* = 7) human donor samples (*p* = 0.0327). **d** No significant mean methylation difference was observed for cg18486102 (*FAIM2*) in AMD (*n* = 30) compared to normal donor RPE cells (*n* = 25) (*p* = 0.1672). **e** Sex-stratified analysis revealed significant differential hypomethylation in males (AMD male *n* = 19, normal male, *n* = 18) (*p* = 0.0276). No significant mean methylation difference was observed in AMD female (*n* = 11) compared to normal female (*n* = 7) human donor RPE cells (*p* = 0.4362). **f** No significant methylation difference was observed in cg26347887 (*EIF2AK3*) in AMD (*n* = 28) compared to human donor samples (*n* = 23) (*p* = 0.4546) **g**–**i**. Sex-stratified results did not reveal significant methylation changes in males or females. **j** No significant mean methylation difference was observed for cg03243226 (*GRIA4*) in AMD (*n* = 30) compared to human donor RPE cells (*n* = 25) (*p* = 0.2053). **k** Sex-stratified analysis did not show significant differential hypomethylation in AMD male (*n* = 18) compared to normal male (*n* = 18) (*p* = 0.8197); however, a significant mean methylation difference was observed in AMD female (*n* = 12) compared to normal female (*n* = 7) (*p* = 0.0279) human donor RPE samples (**l**). All statistical analysis was performed using the Mann-Whitney *U* test (*p ≤ 0.05)

Bisulfite-pyrosequencing analysis of the promoter-based targets cg01560972 (*RIC3*), cg18486102 *(FAIM2)*, cg03243226 (*GRIA4)*, and cg26347887 (*EIF2AK3*) showed inconsistent methylation differences compared with the direction of methylation change in the DNA methylation array (Fig. 4). Pyrosequencing of cg01560972 within the promoter of *RIC3* showed increased mean methylation observed in AMD female samples, reaching statistical significance (*p* = 0.0327) (Fig. 4c). cg18486102 within the promoter region of *FAIM2* showed significantly reduced methylation in males (*p* = 0.0276) (Fig. 4e). cg03243226, within the promoter of *GRIA4*, demonstrated a significant reduction in methylation in females *p* = 0.0279) (Fig. 4l). Bisulfite-pyrosequencing analysis of the promoter-based cg26347887 (*EIF2AK3*) displayed extremely low levels of methylation in the majority of tested samples (≤ 2% methylation) (Fig. 4g–i).

cg26347887 (*EIF2AK3*), cg1560972 (*RIC3*), and cg18486102 (*FAIM2*) were “flagged” by the GapHunter algorithm because they displayed clustered signals that may reflect a genotype-related methylation state [23] (Additional file 2: Figure S4), rendering validation by reproduction of direction of methylation change problematic at these loci (Additional file 2: Figure S5). Clustered *β* value distributions were evident in 74,355 probes in the 450k array data, highlighting that SNPs not routinely excluded from analyses may exert a large effect on methylation states (Additional file 2: Figure S4, Additional file 1: Table S8).

### Site and region-specific DNA methylation differences observed in AMD are not due to global DNA methylation changes

**Fig. 5:**
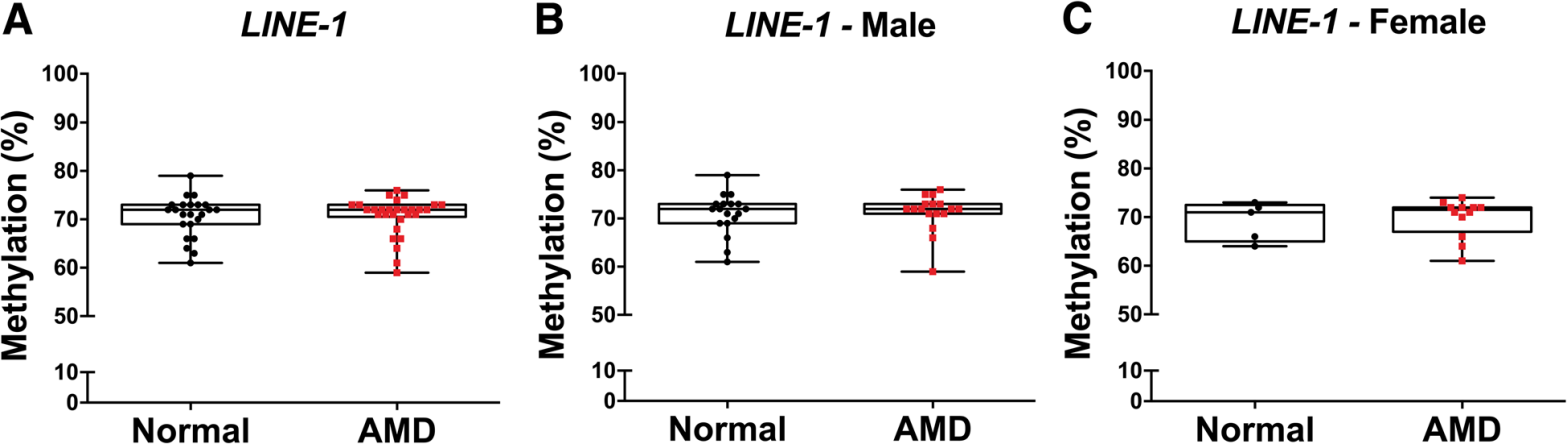
Global methylation analysis of LINE-1 using bisulfite pyrosequencing of AMD RPE cells compared to normals. Mean methylation of LINE-1 compared to normals using bisulfite pyrosequencing. **a** LINE-1 analysis did not identify significant differences between AMD donor RPE cells (*n* = 29) and normal donor RPE cells (*n* = 23) (*p* = 0.8788). Sex-stratified analyses revealed no significant methylation differences in AMD male (*n* = 17) compared to normal male (*n* = 18) (*p* = 0.7730) (**b**) or AMD female (*n* = 12) compared to normal female human RPE cells (*n* = 5) (*p* = 0.8361) (**c**). All statistical analysis was performed using the Mann-Whitney *U* test

A recent study assessing global chromatin accessibility in AMD using Assay for Transposase-Accessible Chromatin using sequencing (ATAC-seq) reported that global reduction of chromatin accessibility occurs in the RPE with early disease [7]. Global DNA-methylation changes have also been identified as a prominent feature of age-related diseases in humans and are associated with genomic instability [26]. Therefore, we analyzed global DNA methylation in RPE samples from AMD donors and normal controls (*n* = 55) (Additional file 1: Table S5) employing the established long interspersed nucleotide element 1 (*LINE-1*) as a proxy element [26]. No significant mean methylation difference was observed in AMD compared to normal human donor samples (Fig. 5). These data imply that site and region-specific DNA methylation differences observed in early and intermediate AMD are not due to global DNA methylation changes.

### Gene expression changes are present in differentially methylated genes *SKI*, *GTF2H4*, and DMR gene *TNXB* in AMD

To prioritize candidate genes for gene expression studies, we investigated individual differentially methylated genes by mining the literature, open access RNA sequencing data, and performed a systematic search for any association with AMD genetic variants (Additional file 1: Table S7). We also performed a systematic search of our differentially methylated CpG loci (Table 2) and DMR (Table 3) for enrichment of histone modifications indicative of gene regulatory functions including H3K4me1 (enhancer signature), H3K27ac (transcription activation enhancer), H3K4me3 (promoter signature), and CTCF (insulator region) using the ENCODE data for different cells and cell lines (www.encodeproject.org, accessed 1 March 2018) (Additional file 1: Table S9 and S10). We noted an enrichment for histone modifications associated with gene regulatory activities at a number of differentially methylated CpG loci and in all DMR in AMD (Additional file 1: Table S9 and S10). Of particular note, both the individual CpG locus and the region surrounding cg18934822 (*SKI*) was enriched for the enhancer signature H3K4me1 (Additional file 2: Figure S6). A more targeted search for genomic regulatory elements in RPE was then performed, using the open access data from Cherry et al. (doi: 10.1101/412361, http://biorxiv.org/cgi/content/short/412361v1, Epigenomic Profiling and Single-Nucleus-RNA-Seq Reveal Cis-Regulatory Elements in Human Retina, Macula and RPE and Non-Coding Genetic Variation), accessed 14 September 2018 (Additional file 1: Table S11). The differentially methylated CpG probe cg26347887 within *EIF2AK3* was found to be within a putative cis-regulatory element (CRE) in RPE/choroid tissue (Additional file 1: Table S11). Finally, we analyzed the ATAC-sequencing peaks identified in RPE by Wang et al. [7] because ATAC-sequencing identifies accessible DNA regions associated with increased gene activity. Differentially methylated CpG probes in AMD cg26347887 (*EIF2AK3*), cg18486102 (*FAIM2*), cg01560972 (*RIC3*), cg26962595 (*STARD10*), cg11897517 (*SMPD2*), and cg04838987 (*EDEM2)* were associated with ATAC sequencing peaks in the RPE (Additional file 1: Table S12)*.*

**Fig. 6:**
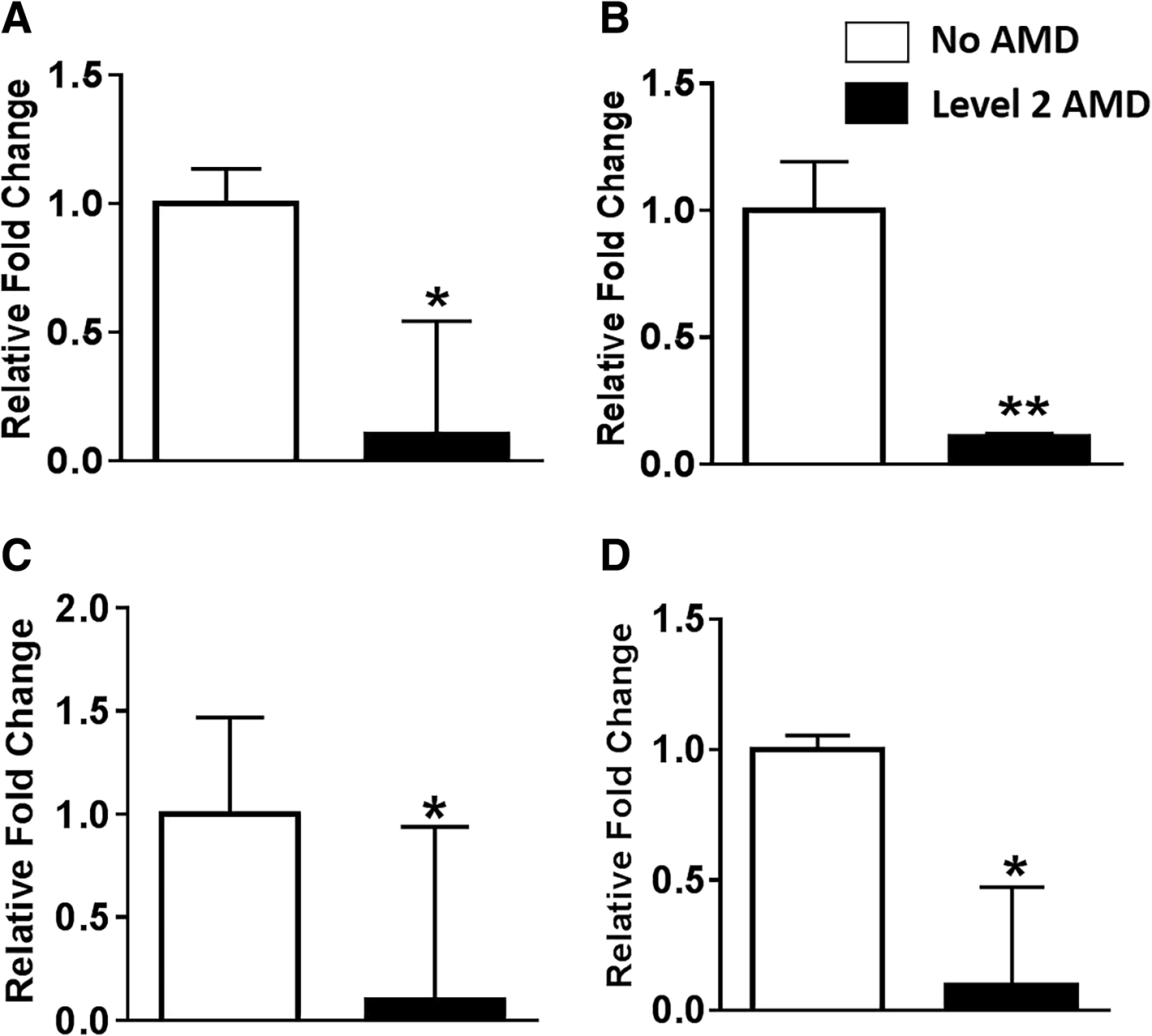
Gene expression of *SKI*, *GTF2H4*, *TNXB*, and *EIF2AK3* and in human donor RPE samples. Relative mRNA quantification of *SKI* (**a**), *GTF2H4* (**b**), *TNXB* (**c**), and *EIF2AK3* (**d**) using RT-qPCR in AMD (*n* = 4) compared to normal (*n* = 3) human donor samples. Significant reduction in *SKI (p = 0.0235)* (**a**), *GTF2H4* (*p* = 0.0174) (**b**), *TNXB* (*p* = 0.05) (**c**), and *EIF2AK3* (*p* = 0.011) (**d**) was present in AMD (*p ≤ 0.05). All statistical analysis was performed using an unpaired Student’s *t* test

We performed RT-qPCR on independent human RPE donor samples from LEITR, Florida, with AMD and normal controls for the most promising target genes including *SKI*, *GTF2H4*, *TNXB*, *RIC3*, and *EIF2AK3*. *GRIA4* was selected because differential *GRIA4* expression has been reported in AMD [37] (Additional file 1: Table S7). Expression of *SKI*, *GTF2H4*, *EIF2AK3*, and *TNXB* was significantly reduced in level 2 AMD compared to normal human donor RPE samples (Fig. 6). Of note, significant expression changes were also present *EIF2AK3* and *GTF2H4* in level 3 AMD RPE cells. No mRNA expression changes were noted for *RIC3*, and *GRIA4* expression levels were very low in AMD (data not shown). Further analysis of our RNA sequencing data [17] confirmed significant decrease in the relative expression of *SKI*, *GTF2H4*, and *TNXB* transcripts in RPE (*n* = 5) donor samples from AMD patients when compared with controls (*n* = 7) (Additional file 2: Figure S7, Additional file 1: Table S2).

## Discussion

Here, we identify novel targets for functional and potential future therapeutic intervention studies, identifying differentially methylated and expressed genes *SKI*, *GTF2H4*, and *TNXB* that regulate disease pathways implicated in AMD. Our findings highlight that DNA methylation changes may influence differential gene expression in early/intermediate AMD. This is an important finding as aberrant DNA methylation in disease may be altered therapeutically [38, 39]. A focus on the RPE is deliberate as the clinical pattern of AMD progression suggests that changes in the RPE precede the dysfunction and death of macular photoreceptors [40] and epigenetic dysfunction of the RPE may drive disease [7].

This is to date the largest DNA methylation study of RPE in AMD with a sample size of relevant ocular tissue significantly higher than those used in other epigenomic studies of AMD (Wang et al., 2018 [7]: RPE—12 AMD, 8 normal; retina—14 AMD, 11 normal; Oliver et al. [12]: retina—9 AMD, 9 normal; Oliver et al. [9]; RPE—3 AMD, 3 normal; retina—9 AMD, 6 normal; Hunter et al. [10]: RPE—10 AMD, 11 normal) and identifying candidate DML that reach genome-wide significance.

*SKI* encodes the SKI proto-oncogene protein that functions as a negative regulator of TGF-β signaling through direct interaction with Smad1-4 [41]. TGF-β signaling has been shown to play a role in RPE cell migration [42]and oxidative stress-induced RPE senescence [43] and modulates complement over-activation, a well-characterized phenomenon of early AMD [44]. Reduced *SKI* in the RPE in AMD could result in increased TGF-β signaling in the RPE, as observed in independent studies [45]. TGF-β stimulation decreases the expression of membrane-bound complement inhibitors on human airway epithelial cells [46] and modulates complement C3 deposition in the RPE [44]. DNA methylation events within *SKI* may contribute to complement dysfunction in AMD, providing potential novel therapeutic approaches to modulate complement deposition in RPE in AMD. Transcriptional changes in *SKI* in AMD RPE have been shown in a previous small study using an Agilent expression array [36] (Additional file 1: Table S7), providing evidence for a need to regulate TGF-β signaling in AMD.

GTF2H4 is a core component of the highly conserved transcription factor II H (TFIIH) basal transcription factor involved in the initiation of transcription, transition from initiation to elongation, and transcription-dependent nucleotide excision repair (NER) of DNA [47]. We observed increased methylation of cg22508626 in the gene body of *GTF2H4* and associated this finding with reduced expression of *GTF2H4* in RPE in AMD. This observation suggests a transcriptional role for exonic methylation, which has been previously posited in alternative splicing regulation [48]. Loss of function mutations in core complex proteins of TFIIH are implicated in rare autosomal recessive human diseases with cell-specific premature aging phenotypes such as xeroderma pigmentosum (DNA repair cancer syndrome), Cockayne syndrome (DNA repair and transcription syndrome/segmental progeria/retinal degeneration), and trichothiodystrophy (DNA repair and transcription syndrome/age-associated pigmentary retinopathy and photoreceptor degeneration) [47, 49]. Reduced TFIIB activity causes impaired tissue handling of oxidative stress lesions that interfere with transcription and the consequent stalled RNA polymerase forms a major trigger for apoptosis [47]. Of relevance, rs3130783 associated with *GTF2H4* has also been identified as an AMD-associated disease locus [32] and several studies have identified *GTF2H4* transcriptional changes in AMD RPE [10, 36]. Reduced expression of *GTF2H4*, as a potential consequence of increased methylation of *GTF2H4* cg22508626, suggests that reduced transcription-dependent DNA repair in the RPE may play a role in AMD.

Our analysis also established the presence of four DMR in AMD RPE (Table 3) including a DMR in *TNXB* containing 15 consecutive CpG probes spanning 424 bp in exon 3 with seven consecutive CpG probes displaying a significant reduction in methylation within this region (Fig. 1). TNX functions in extracellular matrix (ECM) maturation and collagen fibrillogenesis [50], and *TNXB* null mutations result in the connective tissue disorder Ehlers-Danlos Syndrome [51]. We observed a significant reduction in relative *TNXB* mRNA expression in early AMD RPE (Fig. 6). TNX has been shown to localize within the Bruch’s membrane/choroid complex and displays differential expression in AMD patient plasma compared to normal donor samples [52, 53]. Furthermore, multiple GWAS have identified an AMD risk locus in the 6p21.3 region associated with *TNXB* (Additional file 1: Table S7) [30–32] suggesting a role for *TNXB* with altered ECM turnover in the pathogenesis of AMD.

An important factor in our 450k data analysis was the identification of sex as the strongest discriminating factor. Linear interaction modeling was able to normalize for this strong underlying sex-related methylation difference in our bioinformatic analyses. Pyrosequencing analyses were therefore sex-stratified to account for underlying gender-related methylation differences present at some loci. Sex-stratification however reduced the number of samples in the analyses leading to a potential lack of power to detect differential methylation by pyrosequencing at some loci. Therefore, identification of methylation changes in one gender only likely represents under-powering of the technique to detect small methylation changes.

The use of donor tissue with a post-mortem time interval of 24–36 h for DNA methylation analyses is a limitation of this study. However, we made significant attempts to minimize DNA methylation variance linked to postmortem time by extracting all our DNA within a 12 h interval, because DNA methylation variance increases when using tissues with different postmortem collection times, with particularly high variance shown for DNA samples extracted after 72 h [16]. There is evidence to suggest that postmortem times of 24–36 h should not affect the validity of our DNA methylation results significantly [16]. DNA methylation is a very stable modification and epigenetic preservation of methylation patterns has been shown in archeological subjects [54] and in heterogeneous collections of human blood and brain tissue samples [16, 55–57].

## Conclusions

This is the largest genome-wide methylation study reported to date using human RPE cells to show a relationship between differentially methylated loci and regions and early/intermediate AMD. Our data also demonstrates gene expression changes in differentially methylated genes *SKI*, *GTF2H4*, and *TNXB*. Importantly, identified genes *SKI* and *GTF2H4* have not been previously linked to AMD. We also show that the differentially methylated loci and regions identified are not due to global DNA methylation changes. Differentially methylated genes identified in this study are implicated in disease pathways that overlap with known pathological processes. Characterization and understanding of methylation events in AMD may focus efforts on the most relevant pathogenic mechanisms of AMD development. Our findings provide new targets and a rational basis for the design of much-needed therapeutic strategies.

## Additional files

Additional file 1: **Table S1.** Minnesota Grading System (MGS) – based on the AREDS grading system. **Table S2.** Demographic characteristics of all RPE samples used in the study including EWAS discovery cohort, independent sample cohort, and expression studies cohort. **Table S3.** Model parameters used for data analysis. **Table S4.** Bisulfite Pyrosequencing primer sequences. **Table S5.** Comparison of demographic characteristics between AMD cases and normal human donor RPE cells in our EWAS discovery cohort, independent sample cohort and combined cohorts. **Table S6.** RT-qPCR primer sequences. **Table S7.** Candidate Genes: Literature search results. **Table S8.** SNPs associated with candidate gene CpG site. **Table S9.** Histone modifications enriched at differentially methylated CpG loci in various cell types and cell lines represented in the ENCODE data. **Table S10.** Histone modifications enrichment in Differentially Methylated Regions in AMD. **Table S11.** Analysis of differentially methylated CpG loci in relation to proposed Cis-Regulatory Elements (CRE) in the RPE/Choroid from the data of T. Cherry *et al*. (doi**:** https://doi.org/10.1101/412361, http://biorxiv.org/cgi/content/short/412361v1, Epigenomic Profiling and Single-Nucleus-RNA-Seq Reveal Cis-Regulatory Elements in Human Retina, Macula and RPE and Non-Coding Genetic Variation). **Table S12.** Target differentially methylated CpG probes in relation to ATAC-Seq peaks in identified in RPE tissue in Wang et al, 2018^7^. (DOCX 115 kb)

Additional file 2: **Figure S1.** Bar plot of mean *p* values. **Figure S2.** Beta value density distribution plots of Illumina Human Methylation450k BeadChip array before (A) and after (B) SWANN normalization. **Figure S3.** Correlation heatmap showing correlation between AMD (*n*=25) and Normal (*n*=19) donor RPE cells in our Illumina Human Methylation450k BeadChip array dataset. **Figure S4.** Highest ranked probes identified using the GapHunter algorithm. **Figure S5.** Univariate Analysis of *EIF2AK3* and *RIC3* CpG probes from the Illumina Human Methylation450k BeadChip array. **Figure S6.** Enhancer Signature Enrichment (H3K4me1) around differentially methylated cg18934822 within *SKI.* **Figure S7.** Reads Per Kilobase of Transcript per Million Mapped Reads (RPKM) for *SKI, GTF2H4*, *TNXB* and *EIF2AK3* in normal and AMD RPE samples. (PDF 1120 kb)

## Abbreviations

AMD: Age-related macular degeneration
AREDS: Age-related eye disease study
ATAC-seq: Assay for transposase-accessible chromatin using sequencing
CNV: Choroidal neovascularization
CRE: Cis-regulatory element
DML: Differentially methylated locus
DMR: Differentially methylated region
ECM: Extra-cellular matrix
EIF2: Eukaryotic Initiation Factor 2
EIF2AK3: Eukaryotic translation initiation factor 2 alpha kinase
EIF2α: Eukaryotic Initiation Factor 2 alpha
EWAS: Epigenome-wide association study
FAIM2: Fas apoptotic inhibitory molecule 2
FDR: False discovery rate
FOXB1: Forkhead Box B1
GA: Geographic atrophy
GAPH: Glyceraldehyde-3-phosphate dehydrogenase
GTF2H4: General transcription factor IIH subunit H4
GWAS: Genome-wide association study
LEITR: Lions Eye Institute of Transplant and Research
LINE-1: Long interspersed nucleotide element 1
MAF: Minor allele frequency
MDS: Multi-dimensional scaling plot
MGS: Minnesota grading system
NER: Nucleotide excision repair
NHS: National Health Service
PCA: Principal component analysis
PERK: Protein kinase RNA-like ER kinase
qMAMBA: Quantitative methylation analysis of minute DNA amounts after whole bisulfitome amplification
qRT-PCR: Quantitative reverse transcriptase polymerase chain reaction
RIC3: RIC3 acetylcholine receptor chaperone
RPE: Retinal pigment epithelium
SKI: SKI proto-oncogene
SNP: Single nucleotide polymorphism
TFIIH: Transcription factor II H
TGF-β: Transforming growth factor beta
TNXB: Tenascin X
TSS: Transcriptional start site
UPR: Unfolded protein response
UTR: Untranslated region
WGA: Whole-genome amplification

## References

[1] Lim LS, Mitchell P, Seddon JM, Holz FG, Wong TY, Gitter KA (2012). Age-related macular degeneration. Lancet.

[2] Wong WL, Su X, Li X, Cheung CMG, Klein R, Cheng CY (2014). Global prevalence of age-related macular degeneration and disease burden projection for 2020 and 2040: a systematic review and meta-analysis. Lancet Glob Heal.

[3] Ferris FL, Wilkinson CP, Bird A, Chakravarthy U, Chew E, Csaky K (2013). Clinical classification of age-related macular degeneration. Ophthalmology.

[4] Fritsche LG, Chen W, Schu M, Yaspan BL, Yu Y, Thorleifsson G (2013). Seven new loci associated with age-related macular degeneration. Nat Genet.

[5] Clark SJ, Bishop PN (2018). The eye as a complement dysregulation hotspot. Semin Immunopathol.

[6] Porter LF, Black GCM (2014). Personalized ophthalmology. Clin Genet.

[7] Wang J, Zibetti C, Shang P, Sripathi SR, Zhang P, Cano M (2018). ATAC-Seq analysis reveals a widespread decrease of chromatin accessibility in age-related macular degeneration. Nat Commun.

[8] Lee KWK, Pausova Z. Cigarette smoking and DNA methylation. Front Genet. 2013;(4)132:1-11. 10.3389/fgene.2013.00132 PMC3713237 23882278

[9] Oliver VF, Franchina M, Jaffe AE, Branham KE, Othman M, Heckenlively JR (2013). Hypomethylation of the IL17RC promoter in peripheral blood leukocytes is not a hallmark of age-related macular degeneration. Cell Rep.

[10] Hunter A, Spechler PA, Cwanger A, Song Y, Zhang Z, Ying GS (2012). DNA methylation is associated with altered gene expression in AMD. Invest Ophthalmol Vis Sci.

[11] Wei L, Liu B, Tuo J, Shen D, Chen P, Li Z (2012). Hypomethylation of the IL17RC promoter associates with age-related macular degeneration. Cell Rep.

[12] Oliver VF, Jaffe AE, Song J, Wang G, Zhang P, Branham KE (2015). Differential DNA methylation identified in the blood and retina of AMD patients. Epigenetics..

[13] Hutchinson JN, Fagerness J, Kirby A, Reynolds R, Zak A, Gimelbrant A (2014). (Epi)Genetic analyses of age-related macular degeneration: case-control and discordant twin studies. Hum Hered.

[14] Olsen TW, Bottini AR, Mendoza P, Grossniklausk HE. The age-related macular degeneration complex: linking epidemiology and histopathology using the Minnesota Grading System (The Inaugural Frederick C. Blodi Lecture). Trans Am Ophthalmol Soc. 2015;113. PMC5108344 27895380

[15] Olsen TW, Liao A, Robinson HS, Palejwala NV, Sprehe N (2017). The nine-step Minnesota grading system for eyebank eyes with age related macular degeneration: a systematic approach to study disease stages. Investig Ophthalmol Vis Sci.

[16] Rhein M, Hagemeier L, Klintschar M, Muschler M, Bleich S, Frieling H (2015). DNA methylation results depend on DNA integrity-role of post mortem interval. Front Genet.

[17] Kim EJ, Grant GR, Bowman AS, Haider N, Gudiseva HV, Chavali VRM (2018). Complete transcriptome profiling of normal and age-related macular degeneration eye tissues reveals dysregulation of anti-sense transcription. Sci Rep.

[18] Aryee MJ, Jaffe AE, Corrada-Bravo H, Ladd-Acosta C, Feinberg AP, Hansen KD (2014). Minfi: a flexible and comprehensive Bioconductor package for the analysis of Infinium DNA methylation microarrays. Bioinformatics.

[19] Peters TJ, Buckley MJ, Statham AL, Pidsley R, Samaras K, V Lord R (2015). De novo identification of differentially methylated regions in the human genome. Epigenetics Chromatin.

[20] Smyth GK (2005). limma: Linear Models for Microarray Data. Bioinformatics and computational biology solutions using R and Bioconductor.

[21] Phipson B, Maksimovic J, Oshlack A (2015). MissMethyl: an R package for analyzing data from Illumina’s HumanMethylation450 platform. Bioinformatics.

[22] Benjamini Y, Hochberg Y (1995). Controlling the false discovery rate: a practical and powerful approach to multiple testing. J R Stat Soc Ser B.

[23] Andrews SV, Ladd-Acosta C, Feinberg AP, Hansen KD, Fallin MD (2016). “Gap hunting” to characterize clustered probe signals in Illumina methylation array data. Epigenetics Chromatin.

[24] Daca-Roszak P, Pfeifer A, Zebracka-Gala J, Rusinek D, Szybińska A, Jarzab B, et al. Impact of SNPs on methylation readouts by Illumina Infinium HumanMethylation450 BeadChip Array: Implications for comparative population studies. BMC Genomics. 2015;16:1003. 10.1186/s12864-015-2202-0 PMC4659175 26607064

[25] Delaney C, Garg SK, Yung R (2015). Analysis of DNA methylation by pyrosequencing. Methods Mol Biol.

[26] Daskalos A, Nikolaidis G, Xinarianos G, Savvari P, Cassidy A, Zakopoulou R (2009). Hypomethylation of retrotransposable elements correlates with genomic instability in non-small cell lung cancer. Int J Cancer.

[27] Vaissière T, Cuenin C, Paliwal A, Vineis P, Hainaut P, Herceg Z (2009). Quantitative analysis of DNA methylation after whole bisulfitome amplification of a minute amount of DNA from body fluids. Epigenetics.

[28] Livak KJ, Schmittgen TD. Analysis of relative gene expression data using real-time quantitative PCR and the 2-Delta Delta C(T)) Method. Methods (San Diego, Calif.). 2001. 402–408. 10.1006/meth.2001.1262 11846609

[29] Pennington KL, DeAngelis MM (2015). Epigenetic mechanisms of the aging human retina. J Exp Neurosci.

[30] Cipriani V, Leung HT, Plagnol V, Bunce C, Khan JC, Shahid H (2012). Genome-wide association study of age-related macular degeneration identifies associated variants in the TNXB-FKBPL-NOTCH4 region of chromosome 6p21.3. Hum Mol Genet.

[31] Ye Z, Shuai P, Zhai Y, Li F, Jiang L, Lu F (2016). Associations of 6p21.3 region with age-related macular degeneration and polypoidal choroidal vasculopathy. Sci Rep.

[32] Tian L, Kazmierkiewicz KL, Bowman AS, Li M, Curcio CA, Stambolian DE (2015). Transcriptome of the human retina, retinal pigmented epithelium and choroid. Genomics.

[33] Grassmann F, Friedrich U, Fauser S, Schick T, Milenkovic A, Schulz HL (2015). A candidate gene association study identifies DAPL1 as a female-specific susceptibility locus for age-related macular degeneration (AMD). NeuroMolecular Med.

[34] Sasaki M, Harada S, Kawasaki Y, Watanabe M, Ito H, Tanaka H (2018). Gender-specific association of early age-related macular degeneration with systemic and genetic factors in a Japanese population. Sci Rep.

[35] Smith W, Mitchell P, Wang JJ (1997). Gender, oestrogen, hormone replacement and age-related macular degeneration: results from the Blue Mountains Eye Study. Aust N Z J Ophthalmol.

[36] Newman AM, Gallo NB, Hancox LS, Miller NJ, Radeke CM, Maloney MA (2012). Systems-level analysis of age-related macular degeneration reveals global biomarkers and phenotype-specific functional networks. Genome Med.

[37] Whitmore SS, Braun TA, Skeie JM, Haas CM, Sohn EH, Stone EM (2013). Altered gene expression in dry age-related macular degeneration suggests early loss of choroidal endothelial cells. Mol Vis.

[38] Yang X, Lay F, Han H, Jones PA (2010). Targeting DNA methylation for epigenetic therapy. Trends Pharmacol Sci.

[39] Kwa FAA, Thrimawithana TR (2014). Epigenetic modifications as potential therapeutic targets in age-related macular degeneration and diabetic retinopathy. Drug Discov Today.

[40] Bhutto I, Lutty G (2012). Understanding age-related macular degeneration (AMD): relationships between the photoreceptor/retinal pigment epithelium/Bruch’s membrane/choriocapillaris complex. Mol Asp Med.

[41] Pandiyan K, You JS, Yang X, Dai C, Zhou XJ, Baylin SB (2013). Functional DNA demethylation is accompanied by chromatin accessibility. Nucleic Acids Res.

[42] Mitsuhiro MRKH, Eguchi S, Yamashita H (2003). Regulation mechanisms of retinal pigment epithelial cell migration by the TGF-beta superfamily. Acta Ophthalmol Scand.

[43] Yu AL, Fuchshofer R, Kook D, Kampik A, Bloemendal H, Welge-Lüssen U (2009). Subtoxic oxidative stress induces senescence in retinal pigment epithelial cells via TGF-β release. Investig Ophthalmol Vis Sci.

[44] Li Y, Song D, Song Y, Zhao L, Wolkow N, Tobias JW (2015). Iron-induced local complement component 3 (C3) up-regulation via non-canonical transforming growth factor (TGF)-β signaling in the retinal pigment epithelium. J Biol Chem.

[45] Kliffen M, Sharma HS, Mooy CM, Kerkvliet S, de Jong PT (1997). Increased expression of angiogenic growth factors in age-related maculopathy. Br J Ophthalmol.

[46] Gu H, Mickler EA, Cummings OW, Sandusky GE, Weber DJ, Gracon A (2014). Crosstalk between TGF-β1 and complement activation augments epithelial injury in pulmonary fibrosis. FASEB J.

[47] De Waard H, De Wit J, Gorgels TGMF, Van den Aardweg G, Andressoo JO, Vermeij M (2003). Cell type-specific hypersensitivity to oxidative damage in CSB and XPA mice. DNA Repair (Amst).

[48] Gelfman S, Cohen N, Yearim A, Ast G (2013). DNA-methylation effect on cotranscriptional splicing is dependent on GC architecture of the exon-intron structure. Genome Res.

[49] Brooks BP, Thompson AH, Clayton JA, Chan CC, Tamura D, Zein WM (2011). Ocular manifestations of trichothiodystrophy. Ophthalmology.

[50] Chiquet-Ehrismann R, Tucker RP (2011). Tenascins and the importance of adhesion modulation. Cold Spring Harb Perspect Biol.

[51] Schalkwijk J, Zweers MC, Steijlen PM, Dean WB, Taylor G, van Vlijmen IM (2001). A recessive form of the Ehlers-Danlos syndrome caused by tenascin-X deficiency. N Engl J Med.

[52] Nita M, Strzałka-Mrozik B, Grzybowski A, Mazurek U, Romaniuk W (2014). Age-related macular degeneration and changes in the extracellular matrix. Int Med J Exp Clin Res.

[53] Kim HJ, Woo SJ, Suh EJ, Ahn J, Park JH, Hong HK (2014). Identification of vinculin as a potential plasma marker for age-related macular degeneration. Invest Ophthalmol Vis Sci.

[54] Llamas B, Holland ML, Chen K, Cropley JE, Cooper A, Suter CM (2012). High-resolution analysis of cytosine methylation in ancient DNA. PLoS One.

[55] Bär W, Kratzer A, Mächler M, Schmid W (1988). Postmortem stability of DNA. Forensic Sci Int.

[56] El-Harouny E-D, Attalla N, Hasan S, El-Nabi H. The relationship between postmortem interval and DNA degradation in different tissues of drowned rats. J Forensic Sci. 2009;4. Available online at: https://ispub.com/IJFS/4/1/7781. Accessed 6 Mar 2015.

[57] Barrachina M, Ferrer I (2009). DNA methylation of Alzheimer disease and tauopathy-related genes in postmortem brain. J Neuropathol Exp Neurol.

