# Supplementary material for: Whole-genome methylation profiling of the retinal pigment epithelium of individuals with age-related macular degeneration reveals differential methylation of the SKI, GTF2H4, and TNXB genes
Source: Clin Epigenetics. 2019 Jan 14;11:6. doi: 10.1186/s13148-019-0608-2 (PMC6332695; doi:10.1186/s13148-019-0608-2)
Supplement: Supplementary file 1 — Table S1. Minnesota Grading System (MGS) – based on the AREDS grading system. Table S2. Demographic characteristics of all RPE samples used in the study including EWAS discovery cohort, independent sample cohort, and expression studies cohort. Table S3. Model parameters used for data analysis. Table S4. Bisulfite Pyrosequencing primer sequences. Table S5. Comparison of demographic characteristics between AMD cases and normal human donor RPE cells in our EWAS discovery cohort, independent sample cohort and combined cohorts. Table S6. RT-qPCR primer sequences. Table S7. Candidate Genes: Literature search results. Table S8. SNPs associated with candidate gene CpG site. Table S9. Histone modifications enriched at differentially methylated CpG loci in various cell types and cell lines represented in the ENCODE data. Table S10. Histone modifications enrichment in Differentially Methylated Regions in AMD. Table S11. Analysis of differentially methylated CpG loci in relation to proposed Cis-Regulatory Elements (CRE) in the RPE/Choroid from the data of T. Cherry et al. (doi: https://doi.org/10.1101/412361, http://biorxiv.org/cgi/content/short/412361v1, Epigenomic Profiling and Single-Nucleus-RNA-Seq Reveal Cis-Regulatory Elements in Human Retina, Macula and RPE and Non-Coding Genetic Variation). Table S12. Target differentially methylated CpG probes in relation to ATAC-Seq peaks in identified in RPE tissue in Wang et al, 20187. (DOCX 115 kb) [file 13148_2019_608_MOESM1_ESM.docx]

**Supplementary tables**

| **AMD Level** | **MGS/AREDS** |
| --- | --- |
| 1 | Drusen maximum size < 63 μm and total area < 125 μm |
| 2 | (a) Drusen maximum size ≥ 63 μm but < 125 μm, or  (b) Drusen total area ≥ 125 μm, or  (c) RPE abnormalities consistent with AMD |
| 3 | (a) Drusen maximum size ≥ circle 125 μm, or  (b) Drusen maximum size ≥ circle 63 μm and total area > 180 μm and type is soft indistinct, or  (c) Drusen maximum size ≥ 63 μm and total area > 660 μm and type is soft distinct, or  (d) Geographic atrophy (atrophy > 180 μm) within grid but none at centre of macula |
| 4 | (a) Geographic atrophy in central subfield (advanced)  (b) Evidence of Neovascular AMD |

**Table S1. Minnesota Grading System (MGS) – based on the AREDS grading system.**

MGS AMD level 1: no AMD; MGS AMD level 2: Early AMD; MGS AMD level 3: Intermediate AMD; MGS AMD level 4: advanced dry AMD (Geographic atrophy) or choroidal neovascularization.

| **EWAS cohort sample information** | |  |  |  |  |
| --- | --- | --- | --- | --- | --- |
| **Sample_Name** | **Disease_Status** | **Age** | **Gender** | **AMD level (modified MGS)** | |
| CONTROLSAMPLE | TECHNICALcontrol | NA | NA |  |  |
| ETR265 | NORMAL | 61 | M | No AMD |  |
| ETR244 | NORMAL | 73 | F | No AMD |  |
| ETR164 | NORMAL | 65 | M | No AMD |  |
| ETR176 | NORMAL | 74 | M | No AMD |  |
| ETR261 | NORMAL | 83 | F | No AMD |  |
| ETR170 | NORMAL | 70 | M | No AMD |  |
| ETR161 | NORMAL | 66 | M | No AMD |  |
| ETR175 | NORMAL | 71 | M | No AMD |  |
| ETR157 | NORMAL | 85 | M | No AMD |  |
| ETR151 | NORMAL | 72 | M | No AMD |  |
| ETR276 | NORMAL | 78 | M | No AMD |  |
| ETR259 | NORMAL | 74 | M | No AMD |  |
| ETR269 | NORMAL | 72 | F | No AMD |  |
| ETR214 | NORMAL | 82 | F | No AMD |  |
| ETR258 | NORMAL | 75 | M | No AMD |  |
| ETR243 | NORMAL | 75 | F | No AMD |  |
| ETR283 | NORMAL | 83 | F | No AMD |  |
| ETR289 | NORMAL | 74 | F | No AMD |  |
| ETR162 | NORMAL | 66 | M | No AMD |  |
| ETR148 | AMD | 83 | F | Level 2 |  |
| ETR223 | AMD | 66 | M | Level 2 |  |
| ETR173 | AMD | 84 | F | Level 2 |  |
| ETR221 | AMD | 76 | M | Level 2 |  |
| ETR216 | AMD | 79 | M | Level 2 |  |
| ETR213 | AMD | 78 | F | Level 2 |  |
| ETR208 | AMD | 89 | F | Level 2 |  |
| ETR203 | AMD | 76 | M | Level 2 |  |
| ETR292 | AMD | 72 | F | Level 2 |  |
| ETR215 | AMD | 77 | M | level 3 |  |
| ETR220 | AMD | 70 | M | Level 2 |  |
| ETR231 | AMD | 83 | F | Level 3 |  |
| ETR226 | AMD | 68 | M | level 2 |  |
| ETR278 | AMD | 62 | F | level 2 |  |
| ETR171 | AMD | 80 | M | level 2 |  |
| ETR201 | AMD | 76 | M | level 2 |  |
| ETR285 | AMD | 74 | M | level 2 |  |
| ETR224 | AMD | 84 | F | level 3 |  |
| ETR229 | AMD | 59 | M | level 2 |  |
| ETR219 | AMD | 71 | F | level 2 |  |
| ETR204 | AMD | 74 | M | level 2 |  |
| ETR249 | AMD | 50 | M | level 2 |  |
| ETR264 | AMD | 62 | F | Level 2 |  |
| ETR160 | AMD | 73 | M | Level 2 |  |
| ETR210 | AMD | 75 | M | Level 3 |  |
| REPLICATIONCONTROL-102612-40 | CONTROL | 77 | M |  |  |
| **Independent samples for biological replication** | | |  |  |  |
| ETR67 | NORMAL | 74 | M | No AMD |  |
| ETR47 | NORMAL | 70 | M | No AMD |  |
| ETR17 | NORMAL | 82 | M | No AMD |  |
| ETR52 | NORMAL | 78 | M | No AMD |  |
| ETR70 | NORMAL | 75 | F | No AMD |  |
| ETR71 | NORMAL | 65 | F | No AMD |  |
| ETR13 | NORMAL | 75 | M | No AMD |  |
| ETR48 | NORMAL | 59 | M | No AMD |  |
| ETR64 | NORMAL | 69 | M | No AMD |  |
| ETR41 | NORMAL | 67 | F | No AMD |  |
| ETR62 | AMD | 76 | M | level 3 |  |
| ETR93 | AMD | 75 | M | level 2 |  |
| ETR40 | AMD | 71 | M | level 2 |  |
| ETR68 | AMD | 80 | M | level 3 |  |
| ETR16 | AMD | 78 | F | level 2 |  |
| ETR74 | AMD | 76 | F | level 2 |  |
| ETR50 | AMD | 69 | M | level 2 |  |
| **Samples used for RT-qPCR and RNA sequencing** | | |  |  |  |
| **Tissue identification no.** |  |  |  |  | **Experiment Used** |
| 2015-09-5876 | Normal | 81 | F | No AMD | qRT-PCR |
| 2015-09-5968 | Normal | 79 | M | No AMD | qRT-PCR |
| 2015-07-4276 | AMD | 84 | M | level 3 | qRT-PCR |
| 2015-05-4493 | AMD | 78 | M | level 2 | qRT-PCR |
| 2015-07-4330 | AMD | 79 | F | level 3 | qRT-PCR |
| 2015-06-3742 | AMD | 75 | F | level 2 | qRT-PCR |
| TR02 | Normal | 85 | M | No AMD | RNA-Seq |
| TR04 | Normal | 84 | M | No AMD | RNA-Seq |
| TR06 | Normal | 92 | F | No AMD | RNA-Seq |
| TR08 | Normal | 86 | F | No AMD | RNA-Seq |
| TR10 | Normal | 83 | M | No AMD | RNA-Seq |
| TR12 | Normal | 83 | M | No AMD | RNA-Seq |
| TR14 | Normal | 84 | M | No AMD | RNA-Seq |
| TR16 | Normal | 83 | F | No AMD | RNA-Seq |
| TR20 | AMD | 85 | F | level 2 AMD | RNA-Seq |
| TR22 | AMD | 95 | M | level 2 AMD | RNA-Seq |
| TR24 | AMD | 87 | M | level 2 AMD | RNA-Seq |
| TR26 | AMD | 83 | F | level 2 AMD | RNA-Seq |
| TR28 | AMD | 86 | F | level 2 AMD | RNA-Seq |

**Table S2. Demographic characteristics of all RPE samples used in the study including EWAS discovery cohort, independent sample cohort, and expression studies cohort.**

| **Contrast** | **Description** | **Report value** |
| --- | --- | --- |
| Intercept | Global mean of M values | Not reported |
| AMDvsNORMAL | Contrast two conditions taking NORMAL as a reference | logFc, PV, FDR |
| Male vs Female | Contrast two genders taking Female as a reference | logFc, PV, FDR |
| Interaction | Male minus Female using AMDvsNORMAL as a metric | logFc, PV, FDR |
| B39vsB89 | Contrast two batches taking B89 as a reference | logFc, PV, FDR |
| B90vsB89 | Contrast two batches taking B89 as a reference | logFc, PV, FDR |
| B84vsB89 | Contrast two batches taking B89 as a reference | logFc, PV, FDR |

**Table S3. Model parameters used for data analysis.**

Contrasts used between group labels and batch in the linear model based DML analysis. B39, B98, B90 and B84 are prefixing the last two digits of the array Sentrix_ID (position on the array plate) with “B” to distinguish different batches associated with samples in the measurement.

LogFc, Log fold change; PV, *P*-value; FDR, False Discovery Rate.

| **Gene Symbol** | **Primer** | **Primer Sequence** | **Amplicon Location** | **Amplicon Size (bp)** | **Number of CpG’s in Pyrosequencing assay** |
| --- | --- | --- | --- | --- | --- |
| *RIC3* | Forward (*Biotinylated*) | TGGTAAGTGGGGATAGAGA | TSS200/Exon 1 | 81 | 5 |
|  | Reverse | CCCATATTCATCTTTTACTAAAT |  |  |  |
|  | Sequencing | TTTACTAAATCCTATACATTCT |  |  |  |
| *SKI* | Forward | TGTGGAGTGTTTTTAGTTTTTGAT | Intron 1 | 115 | 4 |
|  | Reverse (*Biotinylated*) | TTCCACACCTAAATATCTATCCAA |  |  |  |
|  | Sequencing | TTTGATTGTTATAAAGAAAG |  |  |  |
| *FAIM2* | Forward (*Biotinylated*) | TAGTGGAGATGAGGTTTTGATGT | TSS200/Exon 1 | 125 | 11 |
|  | Reverse | AAACAACCCCAATAAATAAATCC |  |  |  |
|  | Sequencing | CAACCCCAATAAATAAATC |  |  |  |
| *EIF2AK3* | Forward (*Biotinylated*) | GGGTATTTTTTATTGGTAATTG | TSS200/Exon 1 | 102 | 8 |
|  | Reverse | AAACTTTCCCTAATAATCAACAT |  |  |  |
|  | Sequencing | CTTTCCCTAATAATCAACATC |  |  |  |
| *GTF2H4* | Forward (*Biotinylated*) | GGGTATTGTGTATTAGTTAGGTT | Exon 10 | 95 | 4 |
|  | Reverse | CTCTTCCAAAAACCCTCTA |  |  |  |
|  | Sequencing | TTCCAAAAACCCTCTATCC |  |  |  |
| *GRIA4* | Forward (*Biotinylated*) | GATTGGGGTTTGTAGATTT | TS200/Exon 1 | 86 | 4 |
|  | Reverse | CCCTCTCTTAATTTTCCTAAC |  |  |  |
|  | Sequencing | TTCCTAACACCTCTACTTC |  |  |  |
| *LINE-1* | Forward (*Biotinylated*) | TAGGGAGTGTTAGATAGTGGG | LTR | 107 | 6 |
|  | Reverse | CTTCCCAAATAAAACAATACC |  |  |  |
|  | Sequencing | CCAAATAAAACAATACCTC |  |  |  |

**Table S4. Bisulfite Pyrosequencing primer sequences.**

|  |  | **Normal** | **AMD** | ***P-*value*** |
| --- | --- | --- | --- | --- |
| **EWAS Discovery Cohort**  **N=44** | Average Age (Yrs±SD) | 73.6±6.56  (n=19) | 73.2±8.90  (n=25) | 0.997 |
|  | Median Age (Min-Max) | 75 (50-89) | 74 (61-85) | - |
|  | Male (%) | 12 (63%) | 15 (60%) | - |
|  | Female (%) | 7 (37%) | 10 (40%) | - |
|  | Mean ± SD Males | 71.4 ± 6.50 (n=12) | 71.5 ± 8.06 (n=15) | 0.968 |
|  | Mean ± SD Females | 77.4 ± 5.00, (n=10) | 76.8 ± 9.57 (n=10) | 0.876 |
| **Independent Sample Cohort**  **N=17** | Average Age (Yrs±SD) | 71.4±6.75  (n=10) | 75.65±2.93  (n=7) | 0.148 |
|  | Median Age (Min-Max) | 72 (59-82) | 76 (71-80) | - |
|  | Male (%) | 70% (n=7) | 71% (n=5) | - |
|  | Female (%) | 30% (n=3) | 29% (n=2) | - |
|  | Male: Average Age (Yrs±SD) | 72.4±7.41  (n=7) | 75.0±3.32  (n=5) | 0.489 |
|  | Female: Average Age (Yrs±SD) | 69.0±5.29  (n=3) | 77.0±1.41  (n=2) | 0.140 |
| **Combined cohort**  **N=55** | Average Age (Yrs±SD) | 72.2±6.75  (n=25) | 74.673±7.83 (n=30) | 0.229 |
|  | Median Age (Min-Max) | 72 (59-85) | 76 (50-89) |  |
|  | Male (%) | 72% (n=18) | (n=19) | - |
|  | Female (%) | 28% (n=7) | (n=11) | - |
|  | Male: Average Age (Yrs±SD) | 71.6±6.81  (n=18) | 72.61±7.37 (n=19) | 0.665 |
|  | Female: Average Age (Yrs±SD) | 73.8±6.84  (n=7) | 78.21±7.67 (n=11) | 0.242 |

**Table S5. Comparison of demographic characteristics between AMD cases and normal human donor RPE cells in our EWAS discovery cohort, independent sample cohort and combined cohorts.** Mean and standard deviation (SD), median, minimum (min) and maximum (max) values were used to describe the distributions of age (*). *From Unpaired two sample t-test of means.

| *FAIM2* | **Amplicon Size** | 194 |  |  |  |
| --- | --- | --- | --- | --- | --- |
|  |  | **Sequence** (5' -> 3') | **Length** | **Tm** | **Location** |
|  | Forward Primer | AGTTCGTCGAGTCTTTGTCAGA | 22 | 61 | 291-312 |
|  | Reverse Primer | TGGGTCCAGAACAGCAAGC | 19 | 62.2 | 484-466 |
| *RIC3* | **Amplicon Size** | 190 |  |  |  |
|  |  | **Sequence** (5' -> 3') | **Length** | **Tm** | **Location** |
|  | Forward Primer | TTCAGACTGTATCAAGCGTAGGC | 23 | 62 | 702-724 |
|  | Reverse Primer | TGGATCACACGAGGTAACAGAA | 22 | 60.8 | 891-870 |
| *GRIA4* | **Amplicon Size** | 103 |  |  |  |
|  |  | **Sequence** (5' -> 3') | **Length** | **Tm** | **Location** |
|  | Forward Primer | TTCCGAGCAGCGTGCAAATA | 20 | 62.5 | 68-87 |
|  | Reverse Primer | GCATTGGGGCTGGTGTTATGA | 21 | 62.7 | 170-150 |
| *EIF2AK3* | **Amplicon Size** | 80 |  |  |  |
|  |  | **Sequence** (5' -> 3') | **Length** | **Tm** | **Location** |
|  | Forward Primer | ACGATGAGACAGAGTTGCGAC | 21 | 62.1 | 278-298 |
|  | Reverse Primer | ATCCAAGGCAGCAATTCTCCC | 21 | 62.5 | 357-337 |
| *GTF2H4* | **Amplicon Size** | 178 |  |  |  |
|  |  | **Sequence** (5' -> 3') | **Length** | **Tm** | **Location** |
|  | Forward Primer | TATTGGACCGATTGTATGGGCA | 22 | 61.2 | 89-110 |
|  | Reverse Primer | AGCCCTGTACTTTCCTCCTGA | 21 | 62 | 266-246 |
| *SKI* | **Amplicon Size** | 81 |  |  |  |
|  |  | **Sequence** (5' -> 3') | **Length** | **Tm** | **Location** |
|  | Forward Primer | ACTGGAAGGCGAGACCATCT | 20 | 62.5 | 312-331 |
|  | Reverse Primer | AGCACCGAGTTGAGAATCTGC | 21 | 62.4 | 392-372 |
| *TNXB* | **Amplicon Size** | 248 |  |  |  |
|  |  | **Sequence** (5' -> 3') | **Length** | **Tm** | **Location** |
|  | Forward Primer | GTGGTCCAGTATGAGGACACG | 21 | 61.9 | 10753-10773 |
|  | Reverse Primer | CTGGTGGTCACGTCAGTCAC | 20 | 62.4 | 11000-10981 |

**Table S6. RT-qPCR primer sequences**

| Gene Name | Gene Symbol | Function | Study (Reference) | Tissue/Cell Type Examined | Method | Relevance to Candidate Gene | AMD Transcriptome | AMD: Associated SNP |
| --- | --- | --- | --- | --- | --- | --- | --- | --- |
| Tenascin-XB | *TNXB* | Involved in wound healing through matrix-maturation and inhibits cell-migration and speeds up the formation of collagen fibrils. | (58) | Nasal/temporal/Macular Retina and RPE | RNA-Seq | Increased *TNXB* transcription in macular/nasal/temporal retina compared to macular/nasal/temporal RPE/choroid in Non-AMD patients. |  | rs429608 |
|  |  |  | (30) | Peripheral Blood | GWAS | Identified an intronic variant in *TNXB* in AMD compared to Normal patients |  | rs12153855 |
|  |  |  | (31) | Peripheral Blood | GWAS | Identified an intronic variant in *TNXB* significantly associated with neovascular-AMD compared to Normal patients |  |  |
|  |  |  | (36) | Retina/RPE-Choroid | Microarray | Decrease in fold-change of *TNXB* transcription in AMD compared to non-AMD Retina and RPE | Downregulated |  |
|  |  |  | (53) | Plasma | ELISA | Significant decrease in Tenascin-X plasma protein levels compared to Normal patient plasma protein levels. |  |  |
| Ski Proto-Oncogene | *SKI* | Involved in the negative regulation of TGF-β signalling through interaction with Smad-2/-3/-4 and bone morphogenic protein (BMP). | (58) | Nasal/temporal/Macular Retina and RPE | RNA-Seq | Increased *SKI* gene expression in macular retina compared to macular RPE/choroid in Non-AMD patients. |  |  |
|  |  |  | (36) | Retina/RPE-Choroid | Microarray | Increase in fold-change of *SKI* transcription in AMD compared to non-AMD RPE | Upregulated |  |
| General Transcription Factor IIH Subunit 4 | *GTF2H4* | Involved in general transcription. It is a component of the DNA repair factor IIH (TFIIH) core complex which is involved in repair of damaged DNA. | (36) | Retina/RPE-Choroid | Microarray | Increase in fold-change of *GTF2H4* transcription in AMD retina and RPE compared to non-AMD retina and RPE respectively | Upregulated |  |
|  |  |  | (10) | RPE | Affymetrix Exon Array | Increase in fold-change of *GTF2H4* transcription in AMD compared to non-AMD RPE | Upregulated |  |
| Eukaryotic translation initiation factor 2-alpha kinase 3 | *EIF2AK3* | Functions in the unfolded protein response (UPR) through phosphorylation of the eukaryotic translation initiation factor 2 alpha subunit 3 (EIF2α) on serine-52. | (59) | Macular/ Extramacular RPE/Choroid | Microarray | Increase in average *EIF2AK3* expression in normalized AMD compared to non-AMD donor array data | Upregulated |  |
|  |  |  | (36) | Retina/RPE-Choroid | Microarray | Decreased in fold-change of *EIF2AK3* transcription in AMD compared to non-AMD RPE | Upregulated |  |
|  |  |  | (60) | iPSC Human RPE | *In vitro* | Upregulated *EIF2AK3* transcription upon treatment with lipofuscin component N-retinylidene-N-retinylethanolamine (A2E) |  |  |
|  |  |  | (61) | ARPE19 cell line | *In vitro* | ER stress increased PERK (*EIF2AK3*) expression. Propofol treatment attenuated PERK (*EIF2AK3*) expression after induced ER stress in ARPE19 cells |  |  |
|  |  |  | (62) | ARPE19 cell line | *In vitro* | Induced oxidative stress using sodium iodate upregulates PERK protein expression in ARPE19 cells |  |  |
| Fas Apoptotic Inhibitory Molecule 2 | *FAIM2* | Prevents Fas-mediated apoptosis by interaction with the Fas receptor upstream of the Fas-associated death domain (FADD) containing protein | (58) | Nasal/temporal/Macular Retina and RPE | RNA-Seq | Increased FAIM2 gene expression in nasal/temporal/macular regions of the retina compared to RPE/choroid in Non-AMD patients. |  |  |
|  |  |  | (59) | Macular/ Extramacular RPE/Choroid | Microarray | Increase in average FAIM2 expression in normalised AMD compared to non-AMD donor array data | Upregulated |  |
|  |  |  | (17) | Peripheral Retina and RPE/Choroid/Sclera | RNA-Seq | Increase in fold-change of FAIM2 anti-sense transcription in AMD compared to non-AMD patient peripheral retina | Upregulated |  |
|  |  |  | (63) | Peripheral Blood Monocytes | Microarray | Increase in fold-change of FAIM2 transcription in AMD monocytes compared to non-AMD monocytes | Upregulated |  |
|  |  |  | (10) | RPE | Affymetrix Exon Array | Increase in fold-change of FAIM2 transcription in AMD compared to non-AMD RPE | Upregulated |  |
|  |  |  | (64) | Rat Retina/661W photoreceptor cell line | *In vivo/In vitro* | FAIM2 inhibits Fas-mediated apoptosis in the retina. Significant increase in FAIM2 protein expression independent of mRNA change. |  |  |
| Glutamate Ionotropic Receptor AMPA Type Subunit 4 | *GRIA4* | Heteromeric protein complex that acts as a glutamate receptor with ligand-gated ion channel function, principally functioning in excitatory synaptic transmission in the central nervous system. | (58) | Nasal/temporal/Macular Retina and RPE | RNA-Seq | Increased *GRIA4* transcription in macular/nasal/temporal retina compared to macular/nasal/temporal RPE/choroid in Non-AMD patients. |  |  |
|  |  |  | (59) | Macular/ Extramacular RPE/Choroid | Microarray | Increase in average *GRIA4* transcription in normalized AMD compared to non-AMD donor array data | Upregulated |  |
|  |  |  | (37) | Macular RPE/Choroid | Microarray | Decrease in *GRIA4* expression in macula in early AMD macular RPE/choroid compared to non-AMD | Downregulated |  |
|  |  |  | (36) | Retina/RPE-Choroid | Microarray | Decreased in fold-change of *GRIA4* transcription in AMD retina compared to non-AMD retina | Downregulated |  |
| ER Degradation Enhancing Alpha-Mannosidase Like Protein 2 | *EDEM2* | Targets misfolded glycoproteins for N-glycan dependent endoplasmic reticulum-associated degradation (ERAD). | (58) | Nasal/temporal/Macular Retina and RPE | RNA-Seq | Decreased *EDEM2* gene expression in nasal/temporal/macular regions of the retina compared to RPE/choroid in Non-AMD patients. |  |  |
| Brain-Derived Neurotrophic Factor | *BDNF* | In selected central and peripheral nervous system cell populations promotes neuronal growth, maturation and survival in addition to regulating synaptic transmission and plasticity. | (58) | Nasal/temporal/Macular Retina and RPE | RNA-Seq | Increased *BDNF* gene expression in nasal/temporal/macular regions of the retina compared to RPE/choroid in Non-AMD patients. |  |  |
|  |  |  | (10) | RPE | Affymetrix Exon Array | Decrease in fold-change of *BDNF* transcription in AMD compared to non-AMD RPE | Downregulated |  |
|  |  |  | (65) | Human peripheral/macular retina | Microarray | Increased *BDNF* expression in young compared to old non-AMD macular retina |  |  |
|  |  |  | (66) | Human serum | ELISA | *BDNF* levels are increased in AMD compared to non-AMD patient serum independent of AMD-type and sex | Upregulated |  |
| Resistance to inhibitors of cholinesterase 3 | *RIC3* | Chaperone of the 5-hydroxytryptamine (5-HT) type 3 and nicotinic acetylcholine receptor that influences cell surface expression/endoplasmic reticulum assembly of these receptors | (58) | Nasal/temporal/Macular Retina and RPE | RNA-Seq | Decreased RIC3 transcription in macular retina compared to macular RPE/choroid in Non-AMD patients. |  |  |
|  |  |  | (59) | Macular/ Extramacular RPE/Choroid | Microarray | Increase in average RIC3 transcription in normalised AMD compared to non-AMD donor array data | Upregulated |  |
|  |  |  | (67) | RPE/Choroid/Photoreceptors | Microarray | Increase in fold-change of RIC3 transcription in RPE compared to choroidal tissues of non-AMD patients |  |  |
|  |  |  | (63) | Peripheral Blood Monocytes | Microarray | Decrease in fold-change of *RIC3* transcription in AMD monocytes compared to non-AMD monocytes | Downregulated |  |
|  |  |  | (10) | RPE | Affymetrix Exon Array | Decrease in fold-change of *RIC3* transcription in AMD compared to non-AMD RPE | Downregulated |  |

**Table S7. Candidate Genes: Literature search results.**

**Additional references:**

58. Whitmore SS, Wagner AH, DeLuca AP, Drack A V., Stone EM, Tucker BA, et al. Transcriptomic analysis across nasal, temporal, and macular regions of human neural retina and RPE/choroid by RNA-Seq. Exp Eye Res. 2014;129:93–106.

59. Radeke MJ, Peterson KE, Johnson L V., Anderson DH. Disease susceptibility of the human macula: Differential gene transcription in the retinal pigmented epithelium/choroid. Exp Eye Res. 2007;85(3):366–80.

60. Perusek L, Sahu B, Parmar T, Maeno H, Arai E, Le Y-Z, et al. Di-retinoid-pyridinium-ethanolamine (A2E) Accumulation and the Maintenance of the Visual Cycle Are Independent of Atg7-mediated Autophagy in the Retinal Pigmented Epithelium. J Biol Chem. 2015 27;290(48):29035–44.

61. Zhou X, Wei Y, Qiu S, Xu Y, Zhang T, Zhang S. Propofol decreases endoplasmic reticulum stress-mediated apoptosis in retinal pigment epithelial cells. PLoS One. 2016;11(6).

62. Zhang XY, Ng TK, Brelén ME, Wu D, Wang JX, Chan KP, et al. Continuous exposure to non-lethal doses of sodium iodate induces retinal pigment epithelial cell dysfunction. Sci Rep. 2016;6:37279.

63. Grunin M, Hagbi-Levi S-, Rinsky B, Smith Y, Chowers I. Transcriptome Analysis on Monocytes from Patients with Neovascular Age-Related Macular Degeneration. Sci Rep [Internet]. 2016;6(1):29046. Available from: http://www.nature.com/articles/srep29046

64. Pawar M, Busov B, Chandrasekhar A, Yao J, Zacks DN, Besirli CG. FAS apoptotic inhibitory molecule 2 is a stress-induced intrinsic neuroprotective factor in the retina. Cell Death Differ. 2017;24(10):1799–810.

65. Cai H, Fields MA, Hoshino R, Del Priore L V. Effects of aging and anatomic location on gene expression in human retina. Front Aging Neurosci. 2012;4:8.

66. Afarid M, Torabi-Nami M, Nemati A, Khosravi A, Malekzadeh M. Brain-derived neurotrophic factor in patients with advanced age-related macular degeneration. Int J Ophthalmol. 2015;8(5):991–5.

67. Booij JC, van Soest S, Swagemakers SMA, Essing AHW, Verkerk AJMH, van der Spek PJ, et al. Functional annotation of the human retinal pigment epithelium transcriptome. BMC Genomics. 2009;10:164.

| **Probe I.D** | **Gene** | **Illumina CpG Probe β-Value Distribution** | **Infinium Probe Type** | **SNP (rs)** | **Observed Mutation** | **Minor Allele Frequency** | **SNP in Target CpG** | **SNP<10bp (From target CpG)** | **SNP<50bp (From target CpG)** |
| --- | --- | --- | --- | --- | --- | --- | --- | --- | --- |
| cg18486102 | *FAIM2* | Bimodal | I | rs907947157 | C>C/T | T=0.000008 |  |  | Yes |
|  |  |  |  | rs983506931 | C>C/G | C=0.00002 |  |  | Yes |
| cg18934822 | *SKI* | Cloud-like | I | rs537973297 | C>C/T | T=0.0002 | Yes |  |  |
|  |  |  |  | rs141427213 | G>A/G | A=0.0022 |  |  | Yes |
|  |  |  |  | rs897212810 | A>A/G | G=0.00002 |  |  | Yes |
|  |  |  |  | rs181794299 | G>A/G | A=0.0004 |  |  | Yes |
| cg23169512 | *-* | Bimodal | I | rs534789221 | G>C/G | C=0.0002 |  | Yes |  |
|  |  |  |  | rs963144648 | C>C/T | T=0.00007 |  | Yes |  |
|  |  |  |  | rs573669026 | A>A/C | C=0.0002 |  |  | Yes |
| cg22508626 | *GTF2H4* | Cloud-like | II | rs374731704 | G>A/G | A=0.0001 | Yes |  |  |
|  |  |  |  | rs372844726 | A/C/T | T=0.00003 | Yes |  |  |
|  |  |  |  | rs759193868 | C>A/C | A=0.000009 |  | Yes |  |
|  |  |  |  | rs776194626 | C>A/C | G=0.000009 |  | Yes |  |
|  |  |  |  | rs541154633 | C>C/G | A=0.000009 |  |  | Yes |
|  |  |  |  | rs760294963 | G>A/G | A=0.000009 |  |  | Yes |
|  |  |  |  | rs773217507 | T>C/T | C=0.000009 |  |  | Yes |
|  |  |  |  | rs961771337 | G>G/T | N/A |  |  | Yes |
|  |  |  |  | rs772149687 | A>A/G | N/A |  |  | Yes |
| cg01560972 | *RIC3* | Bimodal | I | rs894544923 | G>A/G | A=0.00004 |  | Yes |  |
|  |  |  |  | rs533364177 | C>C/T | T=0.000008 |  | Yes |  |
|  |  |  |  | rs546139073 | C>C/G | N/A |  | Yes |  |
|  |  |  |  | rs34837664 | -/G (insertion) | N/A |  |  | Yes |
|  |  |  |  | rs567662400 | A>A/C | G=0.000008 |  |  | Yes |
| cg04838987 | *EDEM2* | Bimodal | II | rs904409227 | C>C/T | T=0.000008 | Yes |  |  |
| cg11241206 | *BDNF* | Bimodal | I | rs557440317 | C>A/C | N/A | Yes |  |  |
|  |  |  |  | rs56195957 | G>A/G | A=0.0002 |  | Yes |  |
| cg26347887 | *EIF2AK3* | Trimodal | I | rs769588691 | C>C/T | T=0.000008 |  | Yes |  |
|  |  |  |  | rs562774326 | C>C/T | T=0.0002 |  | Yes |  |
|  |  |  |  | rs768715921 | C>C/T | N/A |  | Yes |  |
|  |  |  |  | rs1017167347 | C>C/T | T=0.00003 |  |  | Yes |
|  |  |  |  | rs569246086 | A/C/T | T=0.00002 |  |  | Yes |
|  |  |  |  | rs185561774 | G>C/G | C=0.0006 |  |  | Yes |
| cg03243226 | *GRIA4* | Cloud-like | I | rs895844778 | A>A/G | G=0.00002 |  | Yes |  |

**Table S8. SNPs associated with candidate gene CpG site**

All SNPs (rs) located within or near candidate gene CpG sites identified by the Illumina 450K array and associated beta-value distributions.

| **Probe I.D.** | **Gene** | **Chromosome Coordinate** | **H3K4me1** | | **H3K27ac** | **H3K4me3** | **CTCF** | | **DNAase Hypersensitivity Cluster** | | **Probe Location** |
| --- | --- | --- | --- | --- | --- | --- | --- | --- | --- | --- | --- |
| cg18486102 | *FAIM2* | 12:50297777 | - | - | | Present | - | | Present | TSS200 | |
| cg18934822 | *SKI* | 1:2191402 | Present | - | | - | - | | - | Body | |
| cg23169512 |  | 15:60290666 | Present | - | | - | - | | - | Intragenic | |
| cg22508626 | *GTF2H4* | 6:30879905 | - | - | | - | - | | - | Body | |
| cg01560972 | *RIC3* | 11:8190837 | Present | - | | Present | - | | - | TSS1500 | |
| cg26962595 | *STARD10* | 11:72504889 | - | Present | | Present | Present | | Present | TSS200 | |
| cg11897517 | *SMPD2* | 6:109761938 | - | Present | | - | - | | Present | 5'UTR | |
| cg04838987 | *EDEM2* | 20:33734406 | Present | Present | | Present | - | | - | Body | |
| cg11241206 | *BDNF* | 11:27723128 | Present | - | | Present | - | | - | TSS1500 | |
| cg03611060 |  | 1:59281067 | - | Present | | Present | - | | Present |  | |
| cg26347887 | *EIF2AK3* | 2:88927196 | - | - | | - | | - | Present | TSS200 | |

TSS, Transcription Start Site; 5'UTR, 5'-Untranslated Region; 3'-UTR, 3'-Untranslated Region; Body, Gene Body; Intragenic, Intragenic region, H3K4me1: Enhancer Signature, H3K27ac: Transcription Activation Enhancer, H3K4me3: Promoter Signature, CTCF: Insulator Region.

**Table S9. Histone modifications enriched at differentially methylated CpG loci in various cell types and cell lines represented in the ENCODE data.**

Histone modifications enriched at target CpG loci identified using the ENCODE data within the UCSC genome browser. Data collection was carried out using the layered H3K4me1, layered H3K4me3, layered H3K27ac, CTCF and DNAse clusters. An enrichment for histone modifications with gene regulatory activities is present at a number of differentially methylated CpG loci.

| **Gene** | **CpG Number** | **Chromosome Coordinates** | **DMR Length (bp)** | **H3K4me1** | **H3K27ac** | **H3K4me3** | **CTCF** | **DNAase Hypersensitivity Cluster** | | **DMR Location** |
| --- | --- | --- | --- | --- | --- | --- | --- | --- | --- | --- |
| *FAIM2* | 7 | chr12:50297477-50297945 | 469 | Present | Present | Present | - | | Present | Promoter/Exon 1/Intron 1 |
| *SKI* | 6 | chr1:2190850-2191658 | 809 | Present | - | - | - | | - | Intron 1 |
|  | 3 | chr17:14201680-14201938 | 259 | Present | - | - | - | | - | Intragenic |
| *TNXB* | 15 | chr6:32063835-32064258 | 424 | Present | - | - | - | | - | Exon 3 |

TSS, Transcription Start Site; 5'UTR, 5'-Untranslated Region; 3'-UTR, 3'-Untranslated Region; Body, Gene Body; Intragenic, Intragenic region, H3K4me1: Enhancer Signature, H3K27ac: Transcription Activation Enhancer, H3K4me3: Promoter Signature, CTCF: Insulator Region.

**Table S10. Histone modifications enrichment in Differentially Methylated Regions in AMD.**

Histone modifications enriched at target differentially methylated regions identified from the ENCODE data within the UCSC genome browser. Data collection was carried out using the layered H3K4me1, layered H3K4me3, layered H3K27ac, CTCF and DNAse clusters. An enrichment for histone modifications with gene regulatory activities is present in DMRs in AMD.

| **Probe I.D.** | **Gene** | **CpG Probe Coordinates (Hg19)** | **CpG Probe Coordinates (Hg38)** | **CRE Coordinates (Hg38)** | **Probe distance from CRE (Upstream/Downstream:bp)** | **Tissue Type** |
| --- | --- | --- | --- | --- | --- | --- |
| cg18486102 | *FAIM2* | 12:50297777 | 12:49903994 | 12:49868930-49869654 | Upstream: 34340 | RPE/Choroid |
| cg18934822 | *SKI* | 1:2191402 | 1:2259963 | 1:2255423-2256403 | Upstream: 4540 | RPE/Choroid |
|  |  |  |  | 1:2272050-2272347 | Downstream: 12087 | RPE/Choroid |
|  |  |  |  | 1:2279251-2280199 | Downstream: 19288 | RPE/Choroid |
| cg23169512 |  | 15:60290666 | 15:59998467 | - | - | RPE/Choroid |
| cg22508626 | *GTF2H4* | 6:30879905 | 6:30912128 | 6:30907816-30908332 | Downstream: 3796 | RPE/Choroid |
| cg01560972 | *RIC3* | 11:8190837 | 11:8169290 | - | - | RPE/Choroid |
| cg26962595 | *STARD10* | 11:72504889 | 11:72793844 | 11:72793913-72794349 | Downstream: 69 | RPE/Choroid |
|  |  |  |  | 11:72808427-72808904 | Downstream: 14583 | RPE/Choroid |
| cg11897517 | *SMPD2* | 6:109761938 | 6:109440735 | - | - | RPE/Choroid |
| cg04838987 | *EDEM2* | 20:33734406 | 20:35146603 | 20:35147235-35147603 | Downstream: 632 | RPE/Choroid |
| cg11241206 | *BDNF* | 11:27723128 | 11:27701581 | - | - | RPE/Choroid |
| cg03611060 |  | 1:59281067 | 1:58815395 | 1:58883224-58883728 | Downstream: 67829 | RPE/Choroid |
| cg26347887 | *EIF2AK3* | 2:88927196 | 2:88627678 | 2:88627419-88628477 | **Within Region** | RPE/Choroid |

TSS, Transcription Start Site; 5'UTR, 5'-Untranslated Region; 3'-UTR, 3'-Untranslated Region; Body, Gene Body; Intragenic, Intragenic region,

**Table S11. Analysis of differentially methylated CpG loci in relation to proposed Cis-Regulatory Elements (CRE) in the RPE/Choroid from the data of T. Cherry *et al*. (**doi: **https://doi.org/10.1101/412361,** [**http://biorxiv.org/cgi/content/short/412361v1**](http://biorxiv.org/cgi/content/short/412361v1)**, Epigenomic Profiling and Single-Nucleus-RNA-Seq Reveal Cis-Regulatory Elements in Human Retina, Macula and RPE and Non-Coding Genetic Variation).**

CpG Probe coordinates are shown in both Hg19 and Hg38. Cis-regulatory element coordinates are shown in Hg38. Upstream distance (bp) is calculated using the CpG Probe Coordinate (Hg38) minus the end coordinate of the CRE. Downstream distance (bp) is calculated using the CpG Probe Coordinate (Hg38) minus the CRE start coordinate. ‘Within region’ signifies that the CpG Probe Coordinate (Hg38) is situated within CRE region coordinates. The differentially methylated probe cg26347887 *EIF2AK3* is within a CRE identified in the RPE/choroid tissue. A number of other probes associated with *STARD10*, *EDEM2*, *SKI* and *GTF2H4* are within 5kb of a CRE.

| **Probe I.D.** | **Gene** | | | **CpG Probe Coordinates (Hg19)** | **ATAC-Seq Peak Coordinates (Hg19)** | **CpG Probe distance from ATAC-Seq Peak (Upstream/Downstream:bp)** | | **ATAC-Seq Peak Location** | |
| --- | --- | --- | --- | --- | --- | --- | --- | --- | --- |
| cg18486102 | *FAIM2* | | | 12:50297777 | 12:50260920-50261240 | | Upstream: 36,537 | | 3'UTR |
|  |  | | |  | 12:50262735-50263524 | | Upstream: 34,253 | | 3'UTR |
|  |  | | |  | 12:50297435-50298254 | | **Within region** | | Promoter |
| cg18934822 | *SKI* | | | 1:2191402 | 1:2160649-2160963 | | Upstream: 30,439 | | Promoter |
|  |  | | |  | 1:2162586-2162812 | | Upstream: 28,590 | | Intronic |
|  |  | | |  | 1:2163343-2163764 | | Upstream: 27,638 | | Intronic |
|  |  | | |  | 1:2165753-2166461 | | Upstream: 24,941 | | Intronic |
|  |  | | |  | 1:2171558-2172073 | | Upstream: 19,329 | | Intronic |
|  |  | | |  | 1:2186664-2187889 | | Upstream: 3,513 | | Intronic |
|  |  | | |  | 1:2201691-2202448 | | Downstream: 10,289 | | Intronic |
|  |  | | |  | 1:2210496-2211240 | | Downstream: 19,094 | | Intronic |
|  |  | | |  | 1:2215537-2216224 | | Downstream: 24,135 | | Intronic |
|  |  |  | | | 1:2221994-2222619 | | Downstream: 30,592 | | Intronic |
|  |  | | |  | 1:2230535-2231037 | | Downstream: 39,133 | | Intronic |
|  |  | | |  | 1:2231695-2232156 | | Downstream; 40,293 | | Intronic |
|  |  | | |  | 1:2232180-2232968 | | Downstream: 40,778 | | Intronic |
| cg23169512 |  | | | 5:60290666 | 5:60419706-60420575 | | Downstream: 129,040 | | Intergenic |
| cg22508626 | *GTF2H4* | | | 6:30879905 | 6:30875459-30876316 | | Upstream: 3,589 | | Promoter |
|  |  | | |  | 6:30881474-30882530 | | Downstream: 1,569 | | Exonic,Promoter |
| cg01560972 | *RIC3* | | | 11:8190837 | 11:8190015-8191041 | | **Within region** | | Promoter |
|  |  | | |  | 11:8214046-8214965 | | Downstream: 23,209 | | Intergenic |
|  |  | | |  | 11:8240863-8241676 | | Downstream: 50,026 | | Intergenic |
| cg26962595 | *STARD10* | | | 11:72504889 | 11:72470026-72470903 | | Upstream: 33,986 | | Exonic |
|  |  | | |  | 11:72484266-72484908 | | Upstream: 19,981 | | Intronic |
|  |  | | |  | 11:72492309-72493269 | | Upstream: 11,620 | | 5'UTR |
|  |  | | |  | 11:72504402-72505503 | | **Within region** | | Promoter |
|  |  | | |  | 11:72518059-72519791 | | Downstream: 13,170 | | Intergenic |
| cg11897517 | *SMPD2* | | | 6:109761938 | 6:109761403-109762913 | | **Within region** | | Promoter |
| cg04838987 | *EDEM2* | | | 20:33734406 | 20:33734314-33735788 | | **Within region** | | Promoter |
| cg11241206 | *BDNF* | | | 11:27723128 | 11:27720457-27721503 | | Upstream: 1,625 | | Promoter |
|  |  | | |  | 11:27721730-27722570 | | Upstream: 558 | | Promoter |
|  |  | | |  | 11:27739894-27741101 | | Downstream: 16,766 | | Promoter |
| cg03611060 |  | | | 1:59281067 | - | | - | | - |
| cg26347887 | *EIF2AK3* | | | 2:88927196 | 2:88899445-88900044 | | Upstream: 27,152 | | Intronic |
|  |  | |  | | 2:88926299-88928025 | | **Within region** | | Promoter |

TSS, Transcription Start Site; 5'UTR, 5'-Untranslated Region; 3'-UTR, 3'-Untranslated Region; Body, Gene Body; Intragenic, Intragenic region

**Table S12. Target differentially methylated CpG probes in relation to ATAC-Seq peaks in identified in RPE tissue in Wang *et al*, 2018^7^.**

A number of differentially methylated CpG probes in AMD were associated with ATAC sequencing peaks, including cg18486102 within *FAIM2,* cg01560972 within *RIC3*, cg26962595 within *STARD10*, cg 11897517 associated with *SMPD2*, cg04838987 within *EDEM2* and cg2634788 within *EIF2AK3*.
