# Supplementary material for: Whole-genome methylation profiling of the retinal pigment epithelium of individuals with age-related macular degeneration reveals differential methylation of the SKI, GTF2H4, and TNXB genes
Source: Clin Epigenetics. 2019 Jan 14;11:6. doi: 10.1186/s13148-019-0608-2 (PMC6332695; doi:10.1186/s13148-019-0608-2)
Supplement: Supplementary file 2 — Figure S1. Bar plot of mean p values. Figure S2. Beta value density distribution plots of Illumina Human Methylation450k BeadChip array before (A) and after (B) SWANN normalization. Figure S3. Correlation heatmap showing correlation between AMD (n=25) and Normal (n=19) donor RPE cells in our Illumina Human Methylation450k BeadChip array dataset. Figure S4. Highest ranked probes identified using the GapHunter algorithm. Figure S5. Univariate Analysis of EIF2AK3 and RIC3 CpG probes from the Illumina Human Methylation450k BeadChip array. Figure S6. Enhancer Signature Enrichment (H3K4me1) around differentially methylated cg18934822 within SKI. Figure S7. Reads Per Kilobase of Transcript per Million Mapped Reads (RPKM) for SKI, GTF2H4, TNXB and EIF2AK3 in normal and AMD RPE samples. (PDF 1120 kb) [file 13148_2019_608_MOESM2_ESM.pdf]

Supplementary Figures

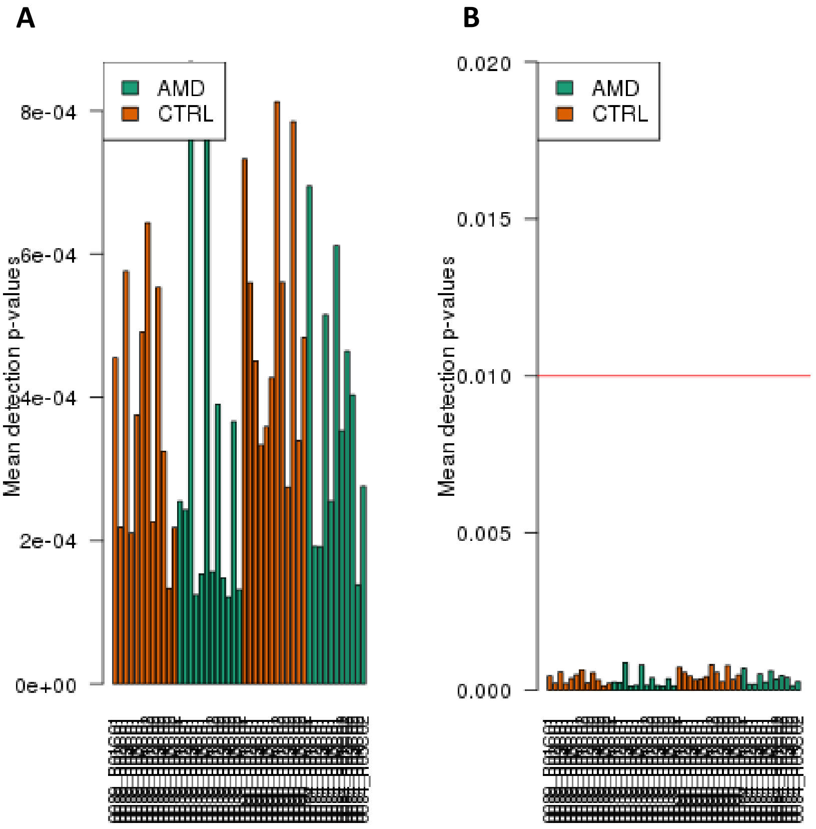

Figure S1 Bar plot of mean *p*-values.

The left panel shows the mean *p*-value of signal quality. A smaller *p*-value is indicative of better signal quality. The right panel is identical to the left panel except that a p-value cut off of 0.01 was applied to "Mean detection p-values" to identify samples of poor quality in our data set.

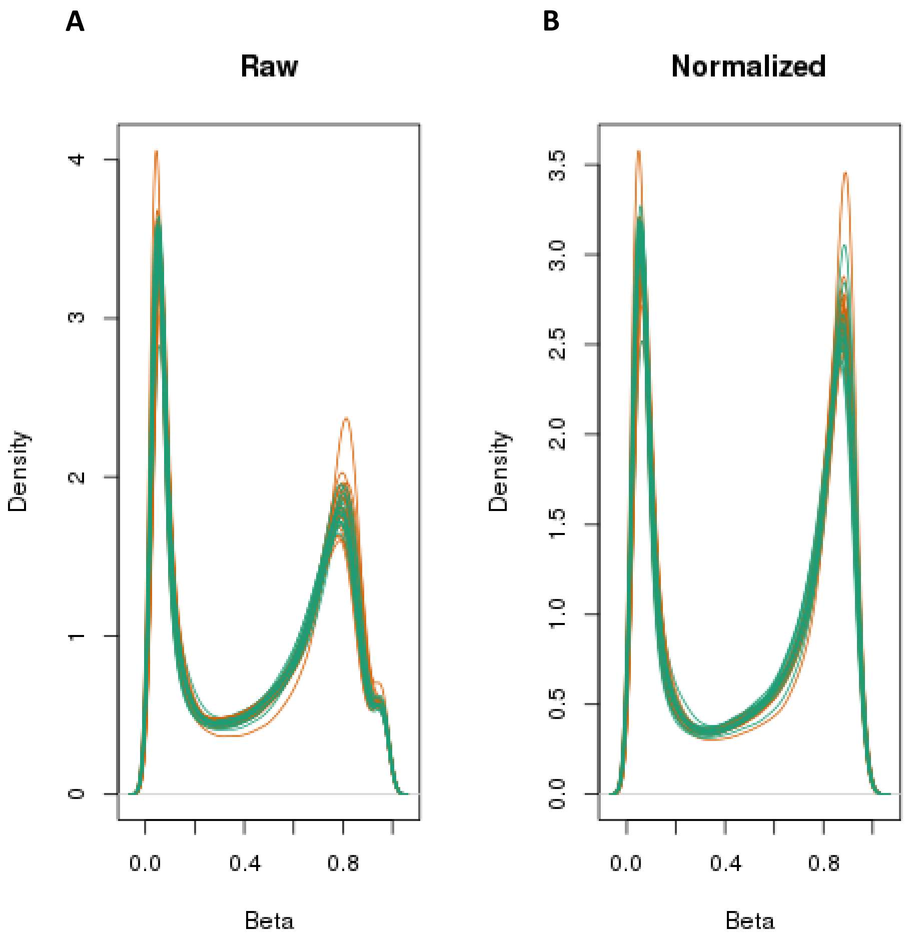

Figure S2 Beta value density distribution plots of Illumina Human Methylation450k BeadChip array before (A) and after (B) SWANN normalization.

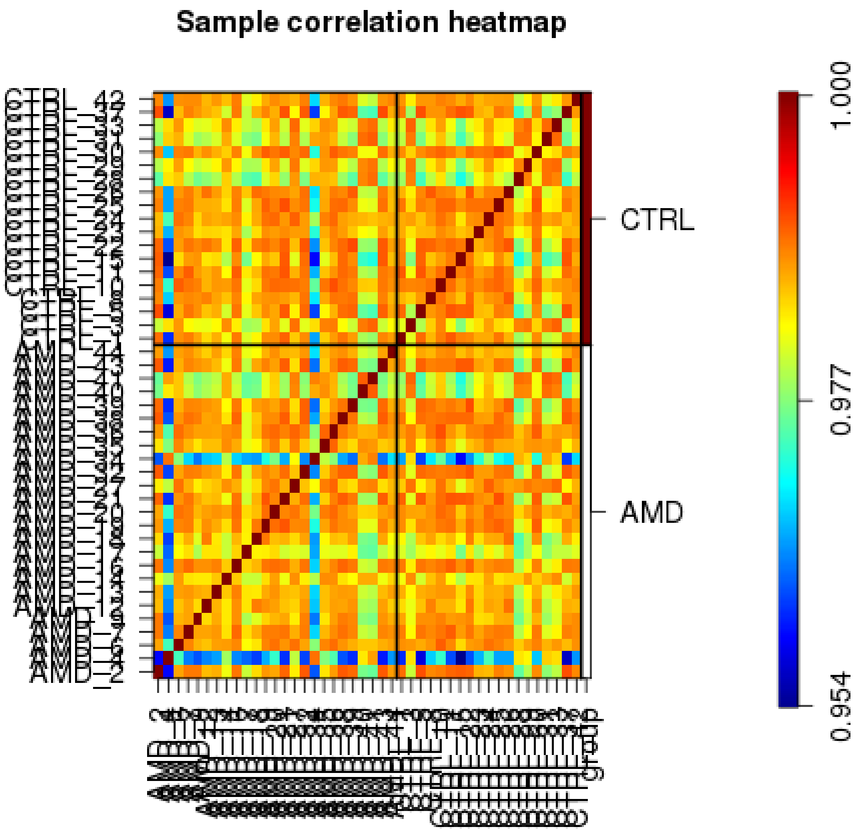

Figure S3 Correlation heatmap showing correlation between AMD (n=25) and Normal (n=19) donor RPE/choroid [illegible]s in our Illumina Human Methylation450k BeadChip array dataset.

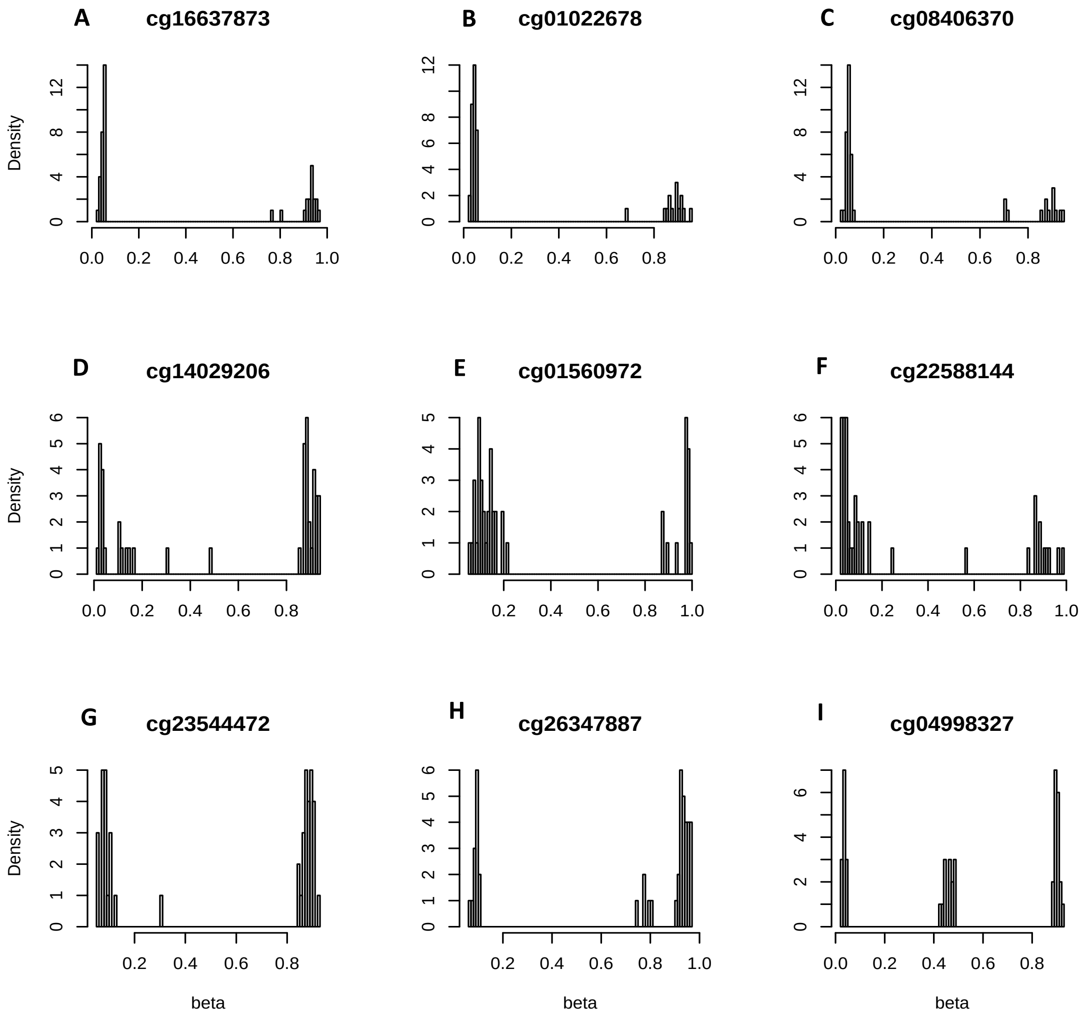

Figure S4 Highest ranked probes identified using the GapHunter algorithm.

Histogram illustrating the presence of discrete beta-value clusters identifying clear gap signals in our Illumina Human Methylation450k BeadChip array dataset. The top gap signals from the dataset are classified as bimodal (A, B, D, E, F, G) and trimodal (C, H, I). All analysis was performed using the 'GapHunter' function in R studio.

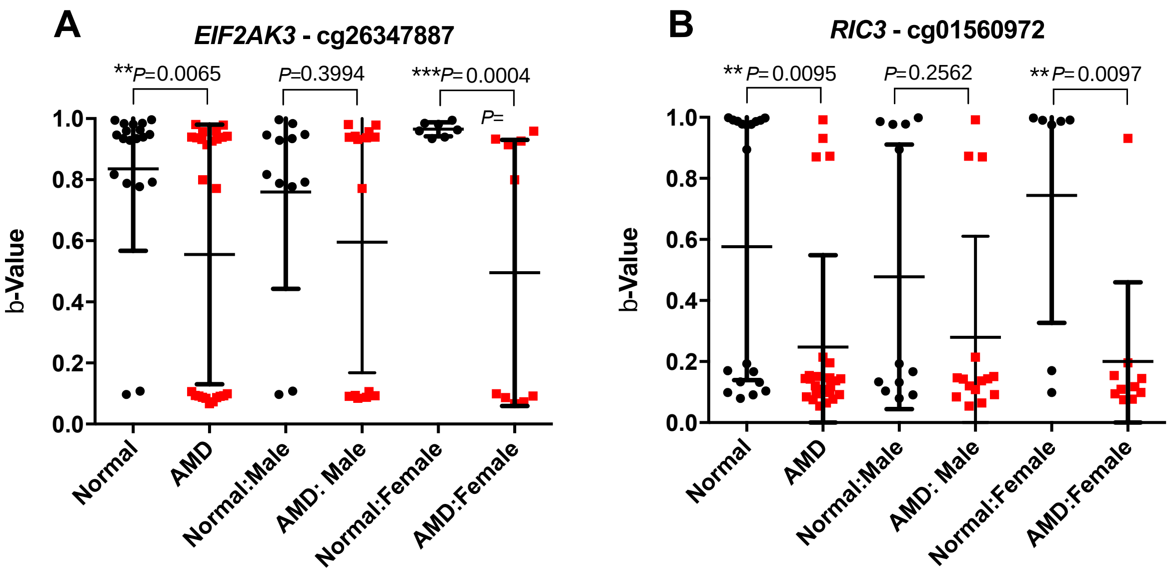

Figure S5 Univariate Analysis of EIF2AK3 and RIC3 CpG probes from the Illumina Human Methylation450k BeadChip array. (A-B) Methylation β-values for AMD (n=25) compared to Normal (n=19) RPE donor cells for locus cg26347887 (*EIF2AK3*) and locus cg01560972 (*RIC3*). Methylation β-values have been stratified by sex for both loci. Significant hypomethylation is observed in AMD compared to normal human RPE donor cells for locus cg26347887 (*EIF2AK3*) (**A**) and locus cg01560972 (*RIC3*) (**B**). Discrete clusters of β-value distributions, usually associated with the presence of SNPs situated in the probe region, are present in both CpG probe loci.

(***P*≤0.01) (*** *P*≤0.001). Mann-Whitney U test was used for statistical analysis between all groups tested.

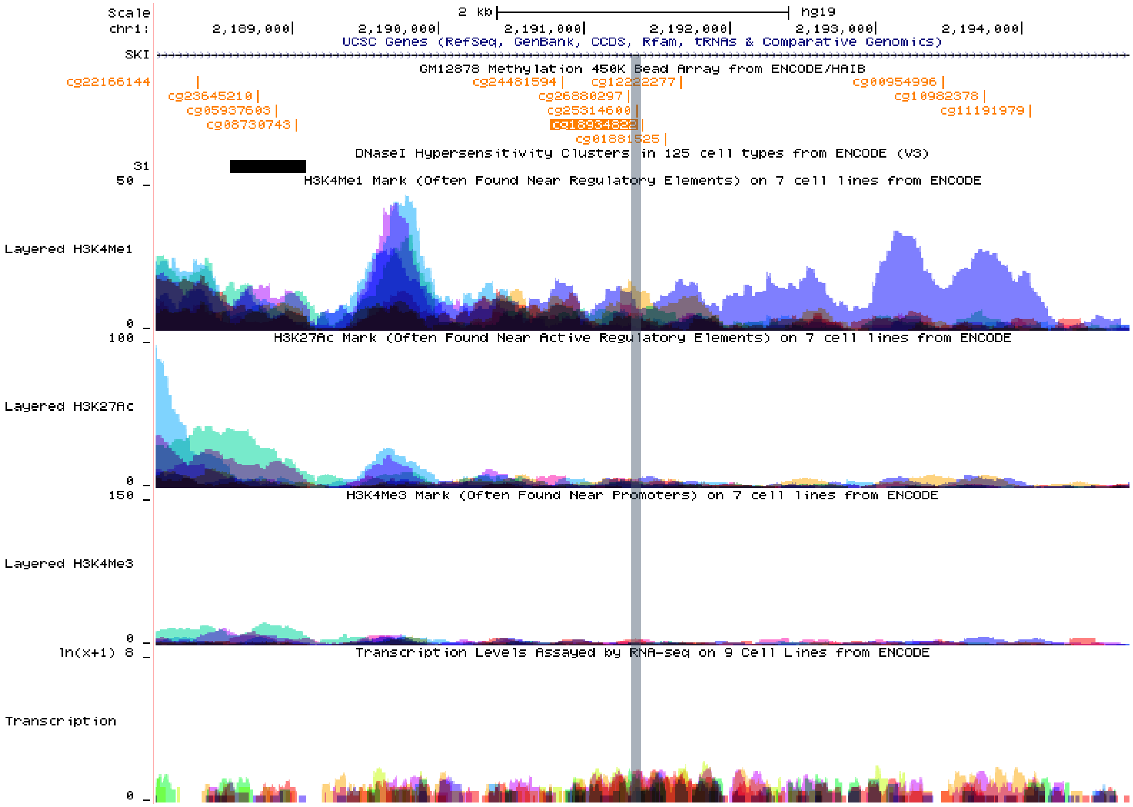

Figure S6 Enhancer Signature Enrichment (H3K4me1) around differentially methylated cg18934822 within *SKI.*

H3K4me1 enrichment shown using ENCODE data for a variety of cell types in UCSC Genome Browser (Hg19).

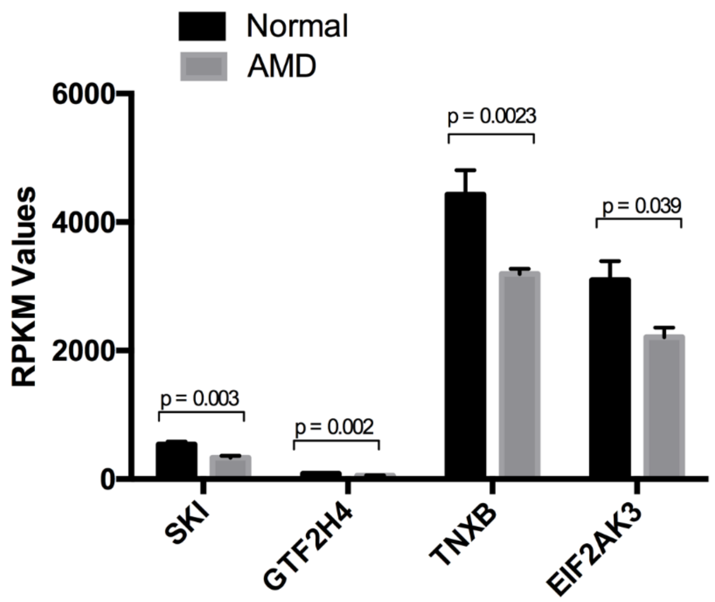

Figure S7 Reads Per Kilobase of Transcript per Million Mapped Reads (RPKM) for *SKI, GTF2H4*, *TNXB* and *EIF2AK3* in normal and AMD RPE samples.

The RPKM values, calculating from the RNA-seq data obtained from RPE-Choroid-Scleral samples from 7 normal donors and 5 AMD donors are shown. Significant reduction in expression of *SKI, GTF2H4*, *TNXB* and *EIF2AK3* is present in AMD. The RPKM values in the RNA-sequencing data presented is proportional to the amount of cDNA present in these tissues.
